# The dorsal tectal longitudinal column (TLCd): a second longitudinal column in the paramedian region of the midbrain tectum

**DOI:** 10.1007/s00429-013-0522-x

**Published:** 2013-03-07

**Authors:** M.-Auxiliadora Aparicio, Enrique Saldaña

**Affiliations:** 1 Department of Cell Biology and Pathology, Medical School, University of Salamanca, 37007 Salamanca, Spain; 2 Neurohistology Laboratory, Neuroscience Institute of Castilla y León (INCyL), University of Salamanca, Laboratorio 6, 37007 Salamanca, Spain; 3 Institute of Biomedical Research of Salamanca (IBSAL), 37007 Salamanca, Spain

**Keywords:** Superior colliculus, Laterodorsal thalamic nucleus, Lateral posterior thalamic nucleus, Visual cortex, Retrosplenial cortex, GABAergic modulation of the thalamus

## Abstract

The tectal longitudinal column (TLC) is a longitudinally oriented, long and narrow nucleus that spans the paramedian region of the midbrain tectum of a large variety of mammals (Saldaña et al. in J Neurosci 27:13108–13116, 2007). Recent analysis of the organization of this region revealed another novel nucleus located immediately dorsal, and parallel, to the TLC. Because the name “tectal longitudinal column” also seems appropriate for this novel nucleus, we suggest the TLC described in 2007 be renamed the “ventral tectal longitudinal column (TLCv)”, and the newly discovered nucleus termed the “dorsal tectal longitudinal column (TLCd)”. This work represents the first characterization of the rat TLCd. A constellation of anatomical techniques was used to demonstrate that the TLCd differs from its surrounding structures (TLCv and superior colliculus) cytoarchitecturally, myeloarchitecturally, neurochemically and hodologically. The distinct expression of vesicular amino acid transporters suggests that TLCd neurons are GABAergic. The TLCd receives major projections from various areas of the cerebral cortex (secondary visual mediomedial area, and granular and dysgranular retrosplenial cortices) and from the medial pretectal nucleus. It densely innervates the ipsilateral lateral posterior and laterodorsal nuclei of the thalamus. Thus, the TLCd is connected with vision-related neural centers. The TLCd may be unique as it constitutes the only known nucleus made of GABAergic neurons dedicated to providing massive inhibition to higher order thalamic nuclei of a specific sensory modality.

## Introduction

The classical view of the midbrain tectum as being exclusively composed of the inferior and superior colliculi (IC and SC, respectively) is challenged by the recent discovery of the tectal longitudinal column (TLC; Saldaña et al. 2007). The TLC is a large, narrow nucleus that spans the midbrain tectum longitudinally, very close to the midline and immediately dorsal to the dorsomedial column of the periaqueductal gray matter (PAGdm). This novel nucleus occupies what has traditionally been considered the most medial region of the deep SC and the most medial region of the IC. The caudal third of the TLC is traversed by the commissure of the IC, and its rostral third is crossed by the rostral portion of the commissure of the SC.

The TLC differs from surrounding nuclei in terms of cytoarchitecture, neural connections and electrophysiological properties. TLC neurons are more homogeneous and significantly smaller than SC and IC neurons (Saldaña et al. 2007). Most TLC neurons have small cell bodies with their main axis oriented rostrocaudally. Scattered throughout the TLC are a few medium-sized neurons, with elongated or triangular cell bodies and more abundant cytoplasm. These cytoarchitectural features have enabled the identification of the TLC in a large variety of mammalian species (Saldaña et al. 2007). The TLC receives projections from various auditory centers, including the superior paraolivary nucleus (Viñuela et al. 2011), the IC (Morest and Oliver 1984; Saldaña and Merchán 1992, 2005; Aparicio et al. 2010) and the primary auditory neocortex (Saldaña et al. 1996). Moreover, most TLC neurons are labeled following injections of retrograde tracers into the ipsilateral superior paraolivary nucleus (Saldaña et al. 2007; Viñuela et al. 2011). Finally, TLC neurons respond vigorously to acoustic stimuli (pure tones or wideband noise), but their responses differ from those of auditory responsive neurons in the IC or the SC (Marshall et al. 2008).

In our initial analysis of the cytoarchitecture of the TLC (Saldaña et al. 2007), we noticed that the territory immediately dorsal to the TLC is occupied by a distinct group of small neurons that differ from the TLC based on cell morphology, size and packing density, and by the fact that they lack connections with auditory structures and do not respond to acoustic stimuli. This led us to conclude that this dorsal territory, which we tentatively called the “dorsal column”, “may constitute another distinct and previously unnoticed columnar nucleus that parallels the TLC and whose further characterization awaits future studies.”

The main purpose of the current investigation is to verify the hypothesis that the dorsal column noticed in 2007 is a distinct nucleus of the mammalian brain. To this end, we have used a large variety of morphological techniques, including neurohistological stains, morphometry, three-dimensional reconstructions, in situ hybridization for vesicular amino acid transporters, and tract-tracing methods to analyze the degree to which the dorsal column differs from its surrounding nuclei (TLC and SC) based on its cytoarchitecture, myeloarchitecture, neurochemistry and neural connections. Because the differences between the dorsal column and the TLC were already established in our original study (Saldaña et al. 2007), this article focuses predominantly on the differences between the dorsal column and the SC, in which it has been traditionally included. As will become evident, our results demonstrate that the dorsal column of the rat differs from both the TLC and the SC and that it is connected with several vision-related centers.

Despite the differences between the TLC and the dorsal column mentioned above, these two nuclei share salient morphological and topographical features. They both posses columnar shapes and span longitudinally the paramedian region of the midbrain tectum. Indeed, they are arranged such that one lies on top of the other. Therefore, the name “tectal longitudinal column”, which we coined for the nucleus described in 2007, seems appropriate for this dorsal column, as well. Accordingly, for the sake of consistency, we suggest ascribing the name “ventral tectal longitudinal column (TLCv)” to the nucleus described by Saldaña et al. (2007), and “dorsal tectal longitudinal column (TLCd)” to the nucleus previously referred to as dorsal column (Table 1). This newly proposed nomenclature will be used throughout the article.

**Table 1:** Nomenclature of the longitudinal columns of the paramedian region of the midbrain tectum

Name used by Saldaña et al. (2007)	New name proposed in this article
Tectal longitudinal column (TLC)	Ventral tectal longitudinal column (TLCv)
Dorsal column	Dorsal tectal longitudinal column (TLCd)

## Materials and methods

### Experimental animals

For the neuroanatomical studies, Sprague–Dawley or Wistar rats of either sex (body weight 190–210 g) were cared for and used in compliance with European Union regulations concerning the use of animals in biomedical research. The experimental procedures were approved and supervised by the Bioethics Committee of the University of Salamanca. For the surgical procedures, including the transcardial perfusion of fixatives, the animals were deeply anesthetized with a mixture of ketamine HCl (80 mg/kg body weight) and xylazine (6 mg/kg body weight) administered intramuscularly.

### Cytoarchitectural analysis

We studied 40–60-μm thick frozen sections or 15-μm thick paraffin-embedded sections of the rat midbrain tectum stained with either 0.25–1 % cresyl violet or with the Giemsa method (Íñiguez et al. 1985). This material was available from the histological collection of our laboratory at the University of Salamanca.

We also used semithin sections of the rat TLCd stained with toluidine blue. To produce these, rats were deeply anaesthetized and perfused with fixative containing 2.5 % glutaraldehyde and 2 % formaldehyde (prepared from freshly depolymerized paraformaldehyde). Vibratome sections (80 μm thick) were postfixed with 1 % osmium tetroxide (OsO_4_), stained with uranyl acetate, dehydrated, cleared with propylene oxide and flat-embedded in EPON 812 resin. Samples containing the TLCd were cut from the section, re-embedded onto blank resin blocks and sectioned with an ultramicrotome at a thickness of 0.5–2 μm. These semithin sections were finally stained with 1 % toluidine blue.

### Myeloarchitectural analysis

We examined 60-μm thick brainstem sections postfixed with OsO_4_. To produce them, we perfused young adult rats with 2.5 % glutaraldehyde and 2 % formaldehyde (prepared from freshly depolymerized paraformaldehyde), dissected the brains and left them in the same fixative overnight. The brains were sectioned with a Vibratome at a thickness of 60 μm. Sections were postfixed in ice-chilled 1 % OsO_4_ and 5 % sucrose in phosphate buffer for about 1 h, dehydrated, cleared with xylene, mounted on slides and coverslipped with Entellan.

We also examined 40-μm thick sections of formaldehyde-fixed brains that were photographed immediately after sectioning unstained and uncleared. For this purpose, we perfused young adult rats with 4 % formaldehyde (prepared from freshly depolymerized paraformaldehyde or from commercial formalin), dissected the brains, cryoprotected them in 30 % sucrose and sectioned them with a freezing microtome. The 40-μm thick sections were allowed to thaw free-floating, transferred to a microscopic slide, coverslipped and photographed unstained in an aqueous medium. The contrast of the micrographs was enhanced uniformly using the Auto Contrast tool of Adobe Photoshop (Adobe, San José, CA, USA) software. For convenience, throughout the article, we will refer to these unprocessed sections as “fresh sections”.

### Reference maps

**Fig. 1:**
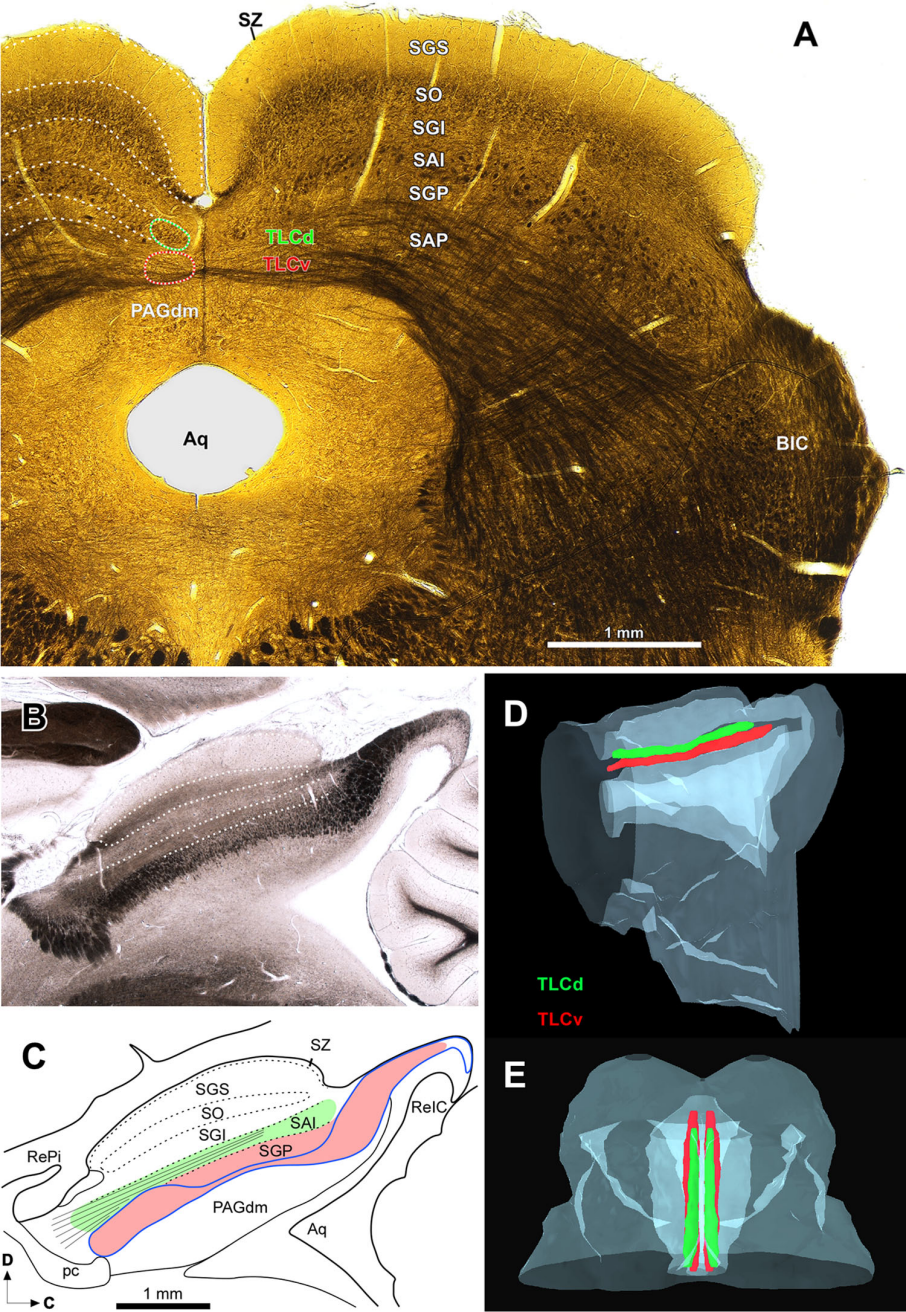
Position and anatomical relationships of the TLCd. **a** Low magnification micrograph of a 60-μm thick coronal section of the rat midbrain tectum postfixed with osmium tetroxide to stain myelinated fibers black. Note the clear stratification of the SC. On the *left side*, the TLCd and TLCv have been outlined in *green* and *red*, respectively. The TLCd occupies the medialmost portion of the classical intermediate white layer of the SC (SAI), defined by the conspicuous bundles of cross-cut longitudinally oriented fibers. **b** Micrograph of a fresh, unstained and uncleared parasagittal section of the rat midbrain tectum through the TLCd and TLCv. In this material, cross-cut fiber fascicles appear dark and stand out over a clear background, thus highlighting the tectal commissures. **c** Schematic drawing of the section depicted in **b**. The *blue line* outlines the continuous fiber fascicle formed by the CoIC and the CoSC. The TLCv has been represented in *light red*, and the TLCd in *green*. The lines that span the TLC and fan out in its rostral pole represent the fascicle of the TLCd (see text). Three-dimensional reconstructions of the rat midbrain, seen from the *left side* of the brain (**d**) or from a point located above and slightly rostral (**e**). The TLCd and the TLCv have been represented in *green* and *red*, respectively. For abbreviations, see list

**Fig. 2:**
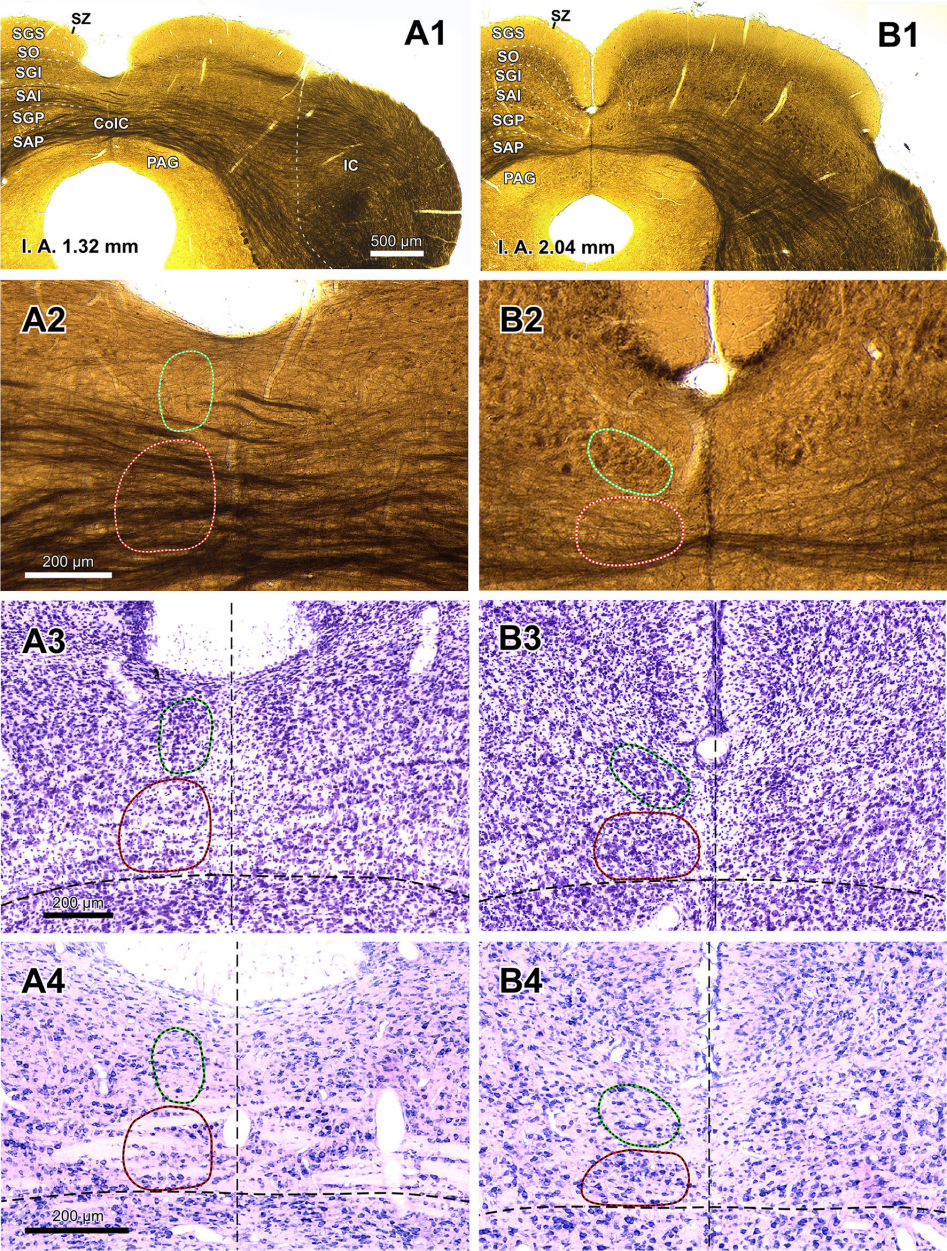

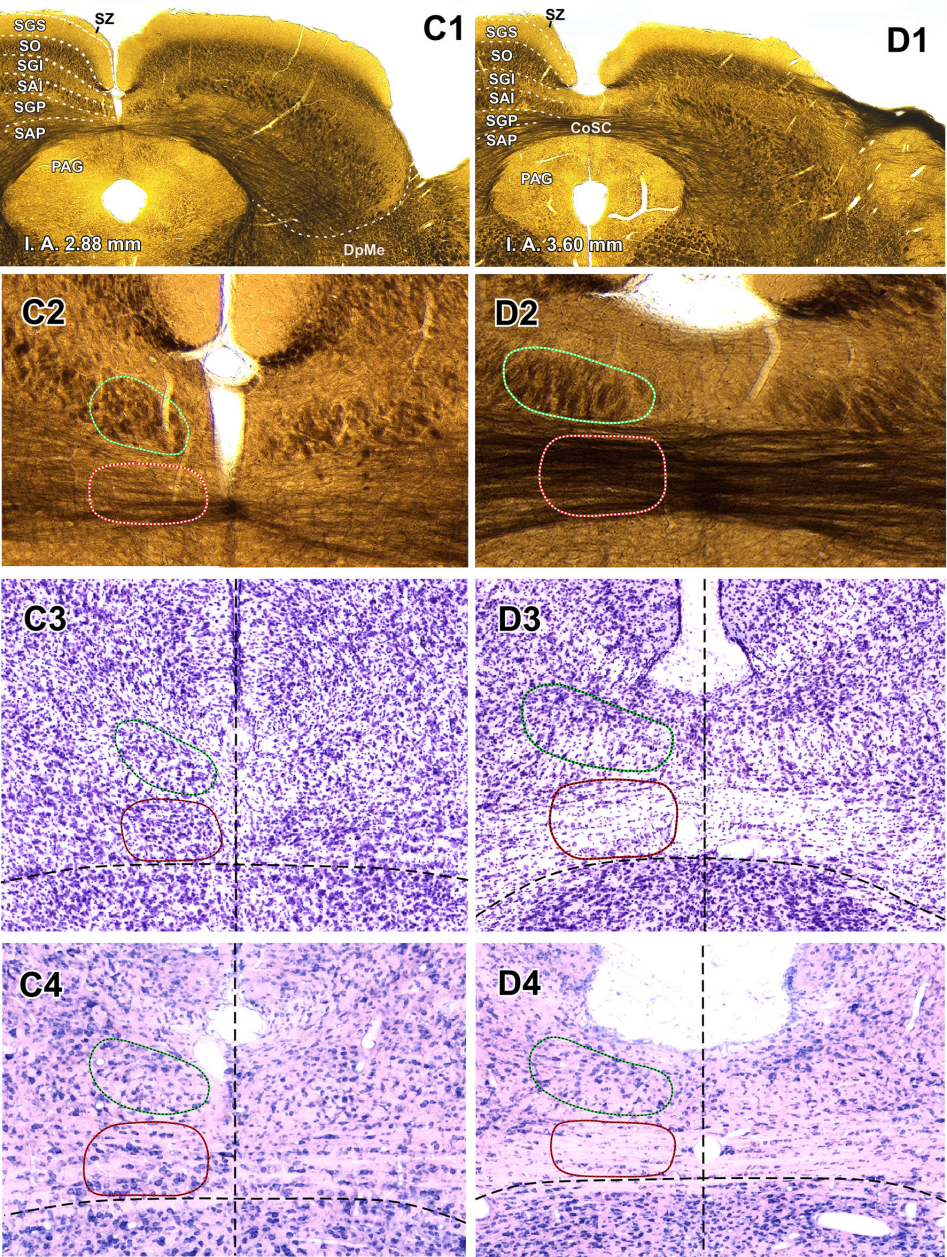
The TLCd in coronal sections. Micrographs of coronal sections through various rostrocaudal levels of the rat TLCd. All four micrographs in any given column correspond to the same rostrocaudal level, whose distance with respect to the coronal interaural plane (IA) is indicated in the *upper panel*. In each column, the first panel (**a1** through **d1**) shows a low magnification micrograph of a 60-μm thick coronal section postfixed with osmium tetroxide. The second picture (**a2** through **d2**) shows at higher magnification the paramedian region of the section depicted above. The third micrograph (**a3** through **d3**) illustrates a 40-μm thick, frozen section stained with the Nissl method, and the fourth micrograph (**a4** through **d4**) shows a 15-μm thick, paraffin-embedded section stained with the Giemsa method. The *left* TLCd has been outlined in *green*, and the *left* TLCv in *red*. The *vertical dashed lines* indicate the *midline*. The *calibration bar* in the first micrograph of *each row* applies to all micrographs in the *same row*. *Calibration bars* uncorrected for shrinkage. For abbreviations, see list

We adopted the well-established parcellation of the mammalian SC, which distinguishes seven alternating layers of white matter and gray matter (reviewed by May 2006). For the delimitation of the layers, we relied on series of thick (60 μm) sections postfixed with OsO_4_ to stain myelinated fibers, and on micrographs of 40-μm thick fresh, unstained sections (see above). Of particular interest was the unequivocal identification of a conspicuous layer of rostrocaudally oriented axons, which, as shown below, is topologically related to the TLCd (e.g., Figs. 1a, 2, 5). In agreement with previous descriptions of the rat SC (Killackey and Erzurumlu 1981; Huerta et al. 1983; Chevalier and Deniau 1984; Matsuyama and Kawamura 1985; Redgrave et al. 1986, 1990; Paxinos et al. 1999; Paxinos and Watson 2005), we will refer to this fiber-rich layer as the intermediate white layer (or stratum album intermediale—SAI). Other authors, however, have considered the layer of longitudinal fibers of rodents as one of the sublaminae or tiers of an enlarged intermediate gray layer (stratum griseum intermediale) (e.g., Wiener 1986; Bickford and Hall 1989; Helms et al. 2004; see May 2006, for additional references).

### Unbiased stereological estimates of neuron numbers

We selected for study three series of coronal sections from our collection of rat brains stained by the Giemsa method (Íñiguez et al. 1985). We chose Giemsa-stained material because this polychromatic technique provides a clear distinction between purple-stained neurons and light blue-stained glial and endothelial cells. This was particularly useful given the small size of most TLCd neurons.

For the stereological estimates of neuron numbers, we followed procedures described elsewhere (Kulesza et al. 2002). Stereo Investigator software (MicroBrightField, Inc.—now MBF Bioscience—, Williston, VT, USA) was used to implement the optical fractionator protocol, which encompasses the optical dissector and fractionator tools (Gundersen 1988; Gundersen et al. 1988). To minimize the coefficient of error, we counted from every fourth section and set the parameters of the optical fractionator so that the sample consisted of at least 250 neurons for each TLCd. This was achieved by sampling from approximately fifteen 400 μm^2^ counting frames per section.

### Three-dimensional reconstructions

In selected cases, we generated 3-D reconstructions of the TLCd using Neurolucida (Version 10) and NeuroExplorer (Version 3) software (MBF Bioscience, Williston, VT, USA). The outline of every other Giemsa-stained coronal section viewed with the 5× (N.A. = 0.12) objective lens of a Leica DMRB microscope was drawn on the computer screen. We then outlined the TLCd (as well as fiduciary marks used to align the drawings of consecutive sections) using a 20× (N.A. = 0.50) objective lens. The sections were aligned with Neurolucida and visualized with NeuroExplorer, rendering visible the surface of the midbrain and the nuclei of interest.

### Morphometry

For comparison of neuronal size across structures, we used a plan apochromatic 100× oil immersion objective (N.A. = 1.4) to measure the maximum diameter of the neuronal cell bodies in semithin, coronal sections through the rostrocaudal center of the rat TLCd. In each section, we measured all neurons with visible nucleoli located in the TLCd, TLCv, and the region of the SC immediately lateral to the TLCd. Neurons chosen for measurement in the SC were limited to those located within 200 μm of the lateral border of the TLCd.

A similar procedure was used to measure the maximum diameters of the cell bodies of neurons of the rat TLCd and SC retrogradely labeled following injections of FluoroGold into the lateral complex of the thalamus (see below). Because of the low number of labeled neurons in the region of the SC adjacent to the TLCd, SC neurons chosen for measurement were limited to those located within 500 μm of the lateral border of the TLCd.

The relative neuronal density across structures was determined using 15-μm thick paraffin-embedded coronal sections of the central third of the rat TLCd stained by the Giemsa method. Using a 100× oil immersion objective (N.A. = 1.4), we counted in each section the neurons included in two square areas of 2,500 μm^2^ randomly placed in each one of the structures analyzed: TLCd, TLCv, and the SC region immediately lateral to the TLCd (within 200 μm of the lateral border of the TLCd).

The morphometric data were analyzed statistically with IBM Predictive Analytics SoftWare (PASW). We first applied the Kolmogorov–Smirnov test to verify whether each set of data followed a normal distribution. The maximum diameter of TLCd, TLCv and SC neurons in non-experimental material was analyzed using Students *t* test for comparisons of two groups, because these values followed normal distributions. The comparisons of neuronal packing density and maximum diameter of FluoroGold labeled neurons were performed with the non-parametric Mann–Whitney test.

### In situ hybridization for vesicular transporters

The description of the distribution of the mRNAs for the vesicular glutamate transporters (VGLUT1 and VGLUT2) and the mRNA for the vesicular inhibitory amino acid transporter (VIAAT) in the paramedian region of the midbrain tectum was based on the same experimental cases used to describe the distribution of these same vesicular transporters in subcortical auditory nuclei (Ito et al. 2011), to which the reader is referred for technical details.

Briefly, deeply anesthetized Long-Evans rats and Swiss-Webster mice were perfused transcardially with 4 % formaldehyde (prepared from freshly depolymerized paraformaldehyde) in 0.1 M phosphate buffer. After cryoprotection in diethylpyrocarbonate (DEPC)-treated 30 % sucrose in PB for 2 days, serial coronal sections were cut at a thickness of 40 μm (for rats) or 30 μm (for mice) with a freezing microtome.

Digoxigenin (DIG)-labeled sense and antisense riboprobes were made from the cDNAs of mouse VGLUT1 (nucleotides of 152–1,085, GenBank accession number NM_182993.2), VGLUT2 (nucleotides of 848–2,044, GenBank accession number NM_080853.2), and VIAAT (nucleotides of 620–1,599, GenBank accession number NM_031782). Sections reacted with sense riboprobes exhibited no signal at all. After extensive washing and acetylation, the sections were incubated for 1 h in a prehybridization buffer, and then for 20 h at 70 °C with the DIG-labeled sense or antisense RNA probe for VGLUT1, VGLUT2, or VIAAT. The sections were then washed, treated with RNase A, incubated with blocking reagent, and finally incubated overnight with alkaline phosphatase-conjugated sheep anti-DIG antibody Fab fragment (Roche Diagnostics, Mannheim, Germany). The bound phosphatase was visualized by a reaction with nitro blue tetrazolium chloride/5-bromo-4-chloro-3-indolyl phosphate toluidine salt in the presence of MgCl_2_. Sections were mounted on glass slides, dehydrated, cleared, and coverslipped.

## Tract-tracing with BDA

We placed single, unilateral injections of BDA (biotinylated dextran amine, 10,000 MW, Molecular Probes, Eugene, OR; 10 % in 0.1 M sodium phosphate buffer, pH 7.4—PB) into the paramedian region of the midbrain tectum. Under stereotaxic guidance, thin glass micropipettes (10–20 μm inner diameter at the tip) loaded with the tracer were inserted vertically into the TLCd or the SC of deeply anaesthetized rats, and the tracer was delivered by iontophoresis using a pulsed 5 μA DC positive current (7 s on/7 s off) for 5–15 min. The current was then stopped and the pipette left in place for an additional 15–20 min prior to withdrawal, in order to prevent leakage of the tracer along the injection tract.

Following a 7-day survival period, the rats were again anesthetized deeply and their brains fixed by transcardial perfusion of buffered 4 % formaldehyde (prepared from freshly depolymerized paraformaldehyde) and 0.1 % glutaraldehyde. After cryoprotection in 30 % sucrose in PB, the brains were cut coronally on a freezing microtome at a thickness of 40 μm. To visualize the tracer, the sections were first processed by the avidin–biotin–peroxidase complex procedure (ABC, Vectastain, Vector Labs, Burlingame, CA, USA) following the manufacturer’s specifications, and then by standard histochemistry for peroxidase, with or without heavy-metal intensification (i.e., Saldaña et al. 2009). For cytoarchitectural reference, every fourth section was counterstained with cresyl violet.

## Tract-tracing with FluoroGold

Glass micropipettes (20–30 μm inner tip diameter) loaded with the retrograde tracer FluoroGold (FG; Fluorochrome Inc., Denver, CO, USA; 4 % in saline) were stereotaxically placed into the lateral complex of the thalamus of deeply anaesthetized rats (*n* = 7). The iontophoretic delivery of the tracer, the survival period, the fixation of the brain by transcardial perfusion, and the sectioning of the brain were as described above.

Initially, we assessed the location of the injection site and labeled neurons by inspecting representative fresh sections on a Leica DMRB microscope under epifluorescence illumination. In selected cases, the FG was rendered permanently visible by immunocytochemistry on free-floating sections, using a rabbit anti-FG primary antiserum (Chemicon International, Inc; Temecula, CA, USA; 1:4,000) followed by biotinylated anti-rabbit immunoglobulin G raised in goat (Vector Labs., Burlingame, CA, USA; 1:50), and then by incubation in the avidin–biotin–peroxidase complex (Vectastain, Vector Labs.) and standard histochemistry for peroxidase, with or without heavy-metal intensification.

## Photography and illustrations

Sections were photographed at high resolution with a Zeiss Axioskop 40 microscope using a Zeiss AxioCam MRc 5 digital camera (Carl Zeiss, Oberkochen, Germany). The brightness and contrast of images were adjusted with Adobe Photoshop software, and the illustrations were arranged into plates using Canvas (ACD Systems of America, Inc., Miami, FL, USA) software.

## Results

### Size and position of the rat TLCd

The rat TLCd is a long, narrow, longitudinally oriented nucleus that spans the rostral two-thirds of the midbrain tectum, very close to the midline and dorsal to the TLCv (Fig. 1). It occupies what has traditionally been considered the most medial portion of the SAI or intermediate white layer of the SC (Figs. 1a, 2, 5). The caudal end of the TLCd is located approximately at the level of the caudal border of the SC and the nucleus extends rostrally all the way to the level of the rostral end of the commissure of the SC (Fig. 1b, c). The TLCd is located immediately dorsal and parallel to most of the TLCv and so is slightly tilted from caudal and dorsal to rostral and ventral (Figs. 1, 2). Unlike the TLCv, the TLCd is not traversed by the commissure of the IC or the commissure of the SC (Figs. 1, 2). The TLCd is covered dorsally by the medial, vertically oriented portion of the superficial layers of the SC, from which it is clearly separated by the most medial portion of the intermediate gray layer (Figs. 1a, 2).

Three-dimensional reconstructions of the rat midbrain tectum illustrate the position, dimensions, columnar shape, and longitudinal orientation of the TLCd, as well as its relationship with the TLCv (Fig. 1d, e). The TLCd is approximately 3.1 mm in length. In caudal coronal sections, it appears as a vertical ovoid structure, 150 μm high and 70–100 μm wide (Fig. 2a). Its mediolateral diameter increases progressively, to reach 350 μm in the most rostral sections (Fig. 2b–d). The number of TLCd neurons on each side is approximately 9,600 (mean = 9,613.50 ± 152.95 SD, *n* = 6), as estimated using methods of unbiased stereology, and it does not differ between the two sides (*t* test, *p* = 0.650; left side 9,646.66 ± 213.12 SD, *n* = 3; right side 9,580.33 ± 98.80 SD, *n* = 3).

### The TLCd is cytoarchitecturally distinct

**Fig. 3:**
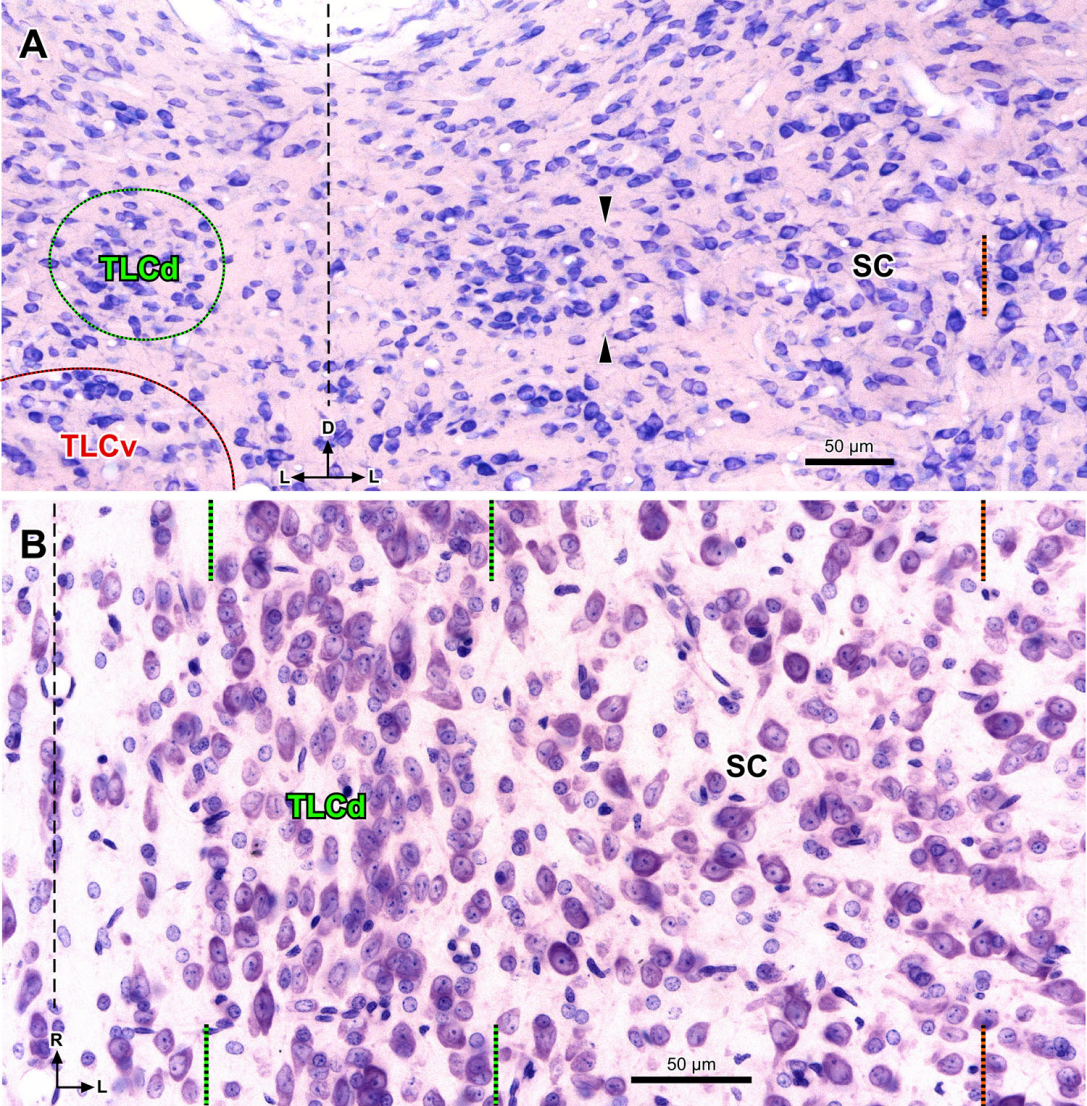
Basic cytoarchitecture of the rat TLCd. **a** Micrograph of a 15-μm thick, paraffin-embedded coronal section stained with the Giemsa method. On the *left side* the TLCd has been outlined in *green*, and the TLCv in *red*. The *black arrowheads* indicate the border between the TLCd and the SC. Note the smaller average size and higher homogeneity and cell packing density of TLCd neurons versus TLCv or SC neurons. The *vertical dashed black line* indicates the *midline*. **b** Micrograph of a 15-μm thick, paraffin-embedded horizontal section through the TLCd and the medial portion of the SC stained with the Nissl method. The *vertical dashed black line* indicates the midline. In this plane of section, the TLCd, delimited by the *green lines*, stands out as a column of small, rostrocaudally oriented and densely packed neurons, which clearly contrast with the much more polymorphic and loosely arranged neurons of the SC. The *orange lines* of both panels indicate the lateral border of the 200 μm wide area of the SC immediately adjacent to the TLCd, where the samples for the quantitative analysis of neuronal size and packing density were taken from. *Calibration bars* uncorrected for shrinkage

**Fig. 4:**
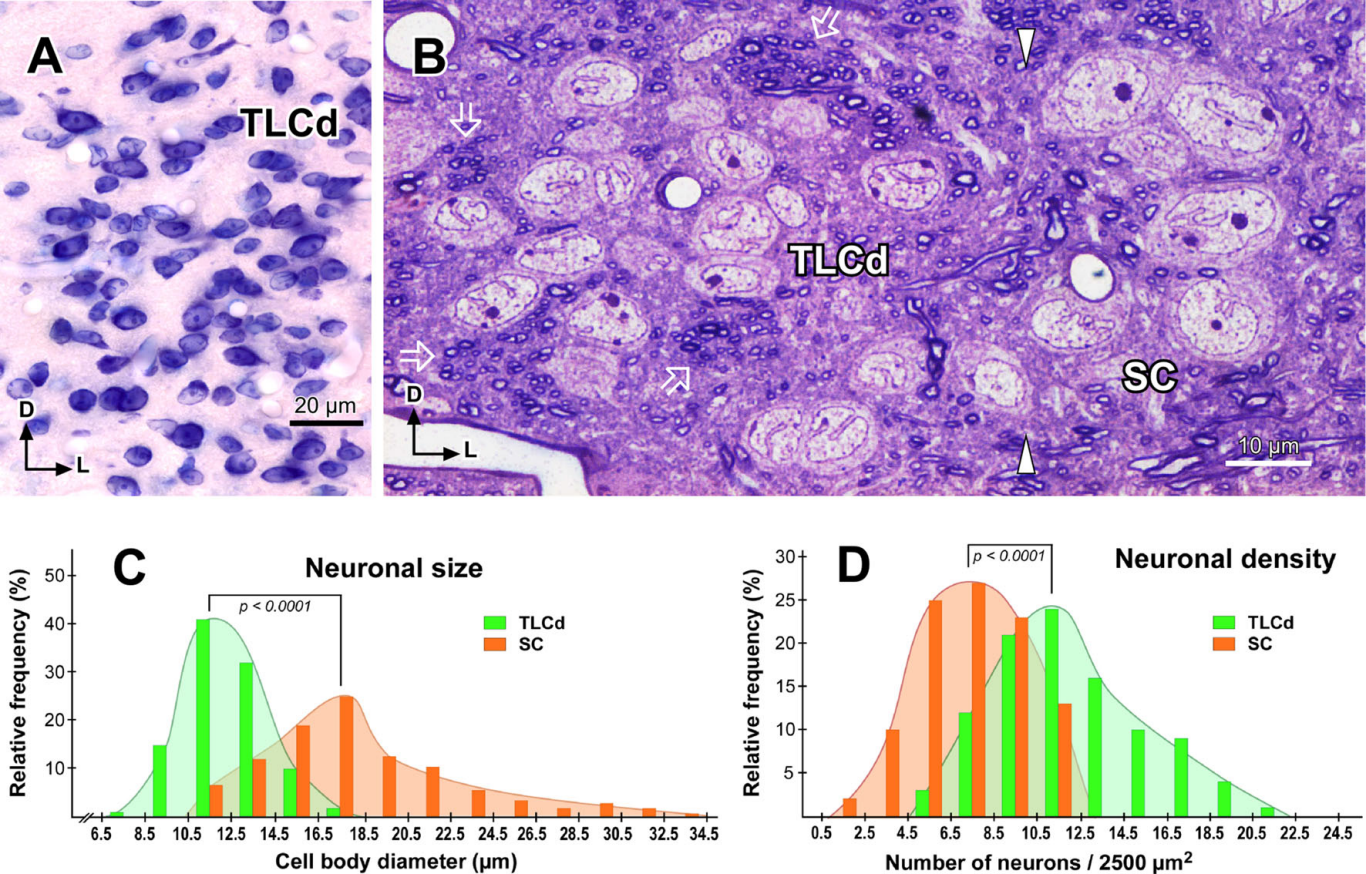
Basic cytoarchitecture and morphometry of the rat TLCd. **a** Detail of the TLCd in a 15-μm thick, paraffin-embedded coronal section stained with the Giemsa method. Note the remarkable homogeneity and predominantly horizontal orientation of the cell bodies. **b** High magnification, digital micrograph of a toluidine blue-stained semithin coronal section through the area of transition between the TLCd and the SC. The border between the two nuclei is indicated by the *white arrowheads*. Note the conspicuous difference between TLCd neurons and the much larger, adjacent SC neurons. The *white open arrows* indicate ill-defined bundles of predominantly thin myelinated axons of the fascicle of the TLCd. *Calibration bars* uncorrected for shrinkage. **c** Histogram of the maximum diameter of the cell body of neurons in the TLCd (*green* mean = 12.24 μm ± 1.78 SD; *n* = 122) and the adjacent SC (*orange* mean = 18.29 ± 4.52 SD, *n* = 185) measured from semithin coronal sections similar to the one depicted in **b**. Measured SC neurons were limited to those located within 200 μm of the lateral border of the TLCd. Note that there is very little overlap between the two populations. SC neurons are generally larger and more heterogeneous than TLCd neurons. **d** Histogram of the neuronal packing density of the TLCd (*green* mean = 7.43 ± 3.73 SD; *n* = 100 square areas) and the adjacent SC (*orange* 7.43 ± 2.49 SD; *n* = 100 square areas). Density is expressed as the number of neurons present in square areas of 2500 μm^2^ of 15 μm thick, coronal paraffin-embedded sections stained by the Giemsa method, similar to the one depicted in Fig. 3a. Sample areas from the SC were limited to those located within 200 μm of the lateral border of the TLCd

Rat TLCd neurons are remarkably homogeneous (Figs. 3, 4). They are distributed uniformly throughout the caudal half of the nucleus (Figs. 2a, b, 3). In the rostral half, neurons tend to concentrate immediately above the fascicle of the TLCd (see below), with few neurons interspersed among the fibers of the fascicle (Fig. 2c, d).

TLCd neurons have small cell bodies, with their main axis oriented rostrocaudally. Consequently, TLCd somata appear elongated (average maximum diameter = 12 μm in paraffin-embedded sections) and are distributed parallel to each other in horizontal sections **(**Fig. 3b**)**, whereas in coronal sections they tend to be rounded or flattened dorsoventrally (Fig. 4a, b) (average maximum diameter = 8–10 μm in paraffin-embedded sections, and 12 μm in resin embedded semithin sections). The nucleus occupies most of the soma and shows several indentations (Figs. 3b, 4a, b). These features of the TLCd contrast with the ventrally adjacent TLCv (Fig. 3a), whose neurons show greater heterogeneity, larger average size (*t* test, *p* < 0.0001; TLCd, *n* = 122; TLCv, *n* = 111) and lower packing density (*t* test, *p* < 0.0001; TLCd, *n* = 100; TLCv, *n* = 100; *n* here refers to number of squares where cells were counted, see Methods). TLCd neurons also contrast with the laterally adjacent SC, whose neurons are much larger (*t* test, *p* < 0.0001; TLCd, *n* = 122; SC, *n* = 185), less densely packed (*t* test, *p* < 0.0001; TLCd, *n* = 100; SC, *n* = 100) and more heterogeneous in size, shape and orientation **(**Figs. 3a, b, 4b–d**)**. Indeed, the transition between the TLCd and the SC is characterized by an abrupt change in the size, shape, orientation and packing density of the neurons (Figs. 3a, b, 4b).

### The TLCd is myeloarchitecturally distinct

**Fig. 5:**
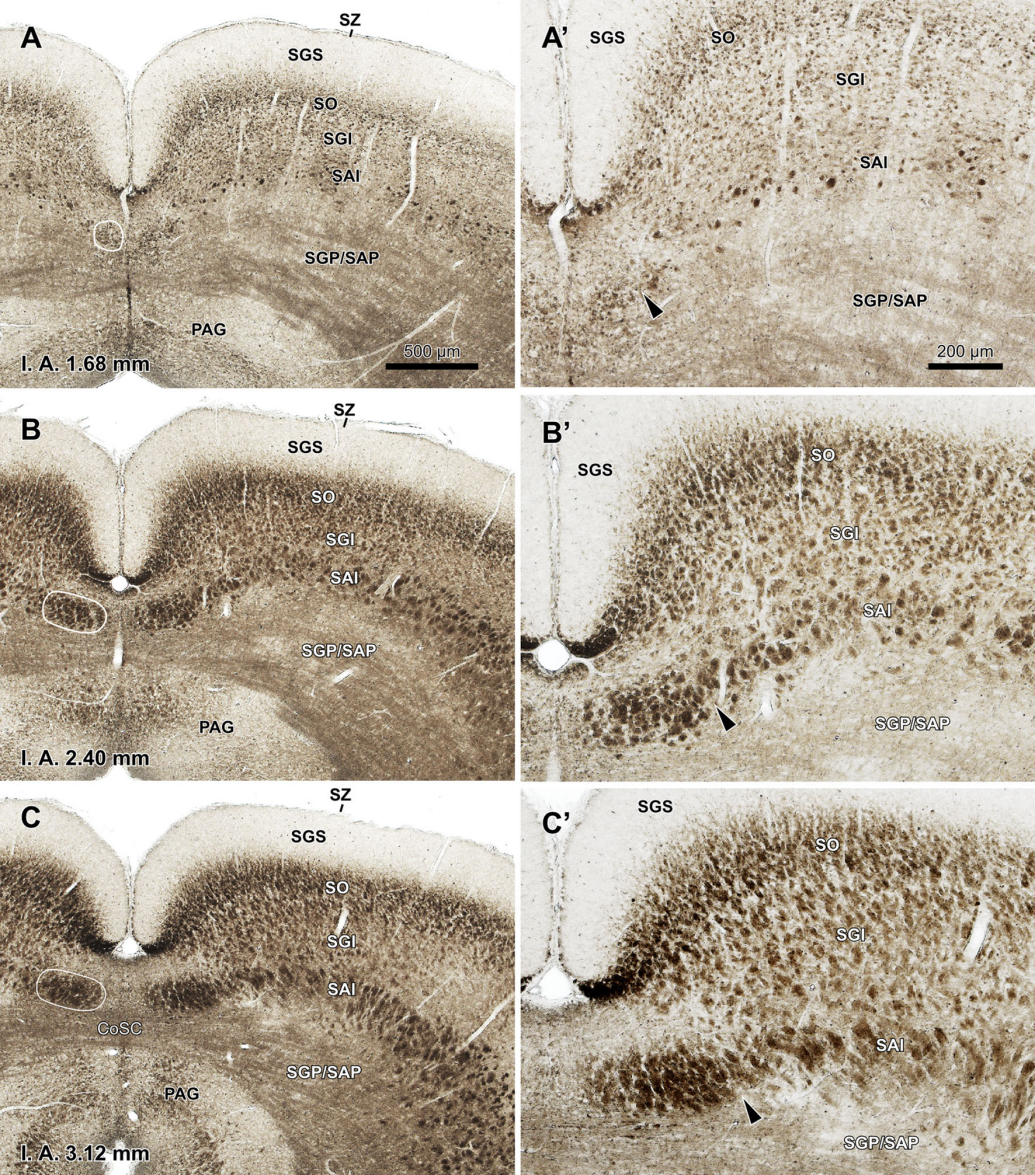
Basic myeloarchitecture of the rat TLCd. **a**–**c** Micrographs of fresh unstained and uncleared coronal sections through three rostrocaudal levels of the midbrain tectum. Each micrograph is paired on the right with a higher magnification of the TLC and the adjacent SAI (**a**′–**c**′). Cross-cut axons appear dark, thus delineating the TLCd, the SAI and the optic layer of the SC (SO). The fascicle of the TLCd has been outlined in *white* on the *left side*. The *black arrowheads* indicate the limit between the fascicle of the TLCd and the SAI. At caudal levels (**a**, **a**′), the TLCd contains individual axons that are clearly discernable from the bundles of cross-cut fibers of the SAI. At central (**b**, **b**′) and rostral levels (**c**, **c**′) the fascicle of the TLCd appears as a distinct darker structure made of cross-cut fiber bundles that are on average thinner and more tightly packed than those of the SAI. The interaural (IA) coordinate of each plane is indicated at the *bottom* of the micrographs. *Calibration bar* in **a** applies also to **b**, **c**. *Calibration bar* in **a**′ applies also to **b**′, **c**′. For abbreviations, see list

Figure 5 shows micrographs of three 40-μm thick fresh, unstained coronal sections through different rostrocaudal levels of the midbrain tectum (see “Materials and methods”). In this type of material, cross-cut myelinated fibers are particularly opaque to light and appear dark-brown over a much clearer background. Although both the TLCd and the SAI are characterized by the presence of rostrocaudally oriented, cross-cut bundles of axons, the two nuclei exhibit a very different appearance. In caudal sections, the SAI contains well-defined bundles of cross-cut myelinated axons, whereas the TLCd contains transversally cut fibers that do not aggregate into bundles (Fig. 5a, a′). In more rostral sections, the two nuclei appear to contain bundles of cross-cut fibers, but the TLCd appears darker than the SAI, which indicates that it contains a higher concentration of longitudinally oriented myelinated axons (Fig. 5b, b′, c, c′). Moreover, the fiber bundles that span the TLCd are thinner, more densely packed and less sharply defined than those of the SAI. This arrangement is compatible with the dense packing of TLCd cell bodies, which forces the longitudinally oriented axons to occupy the narrow spaces left among the neurons (Fig. 4b). In contrast, the thicker, better defined and much more scattered bundles of cross-cut axons of the SAI are compatible with its lower neuronal density. Detailed examination of thick sections postfixed with OsO_4_ and semithin sections stained with toluidine blue further reveal that the longitudinal axons of the TLCd tend to be predominantly thin, whereas the bundles of the SAI consist of fibers of heterogeneous thickness (not shown).

These results indicate that the TLCd contains abundant axons that span the nucleus longitudinally, and which seem to enter or exit the TLCd through its rostral pole. Because these axons differ from those of the intermediate white layer in their caliber and their pattern of aggregation into bundles, they likely collectively represent an anatomical structure separate from the SAI, which will be referred to as the fascicle of the TLCd (Figs. 1c, 2, 5).

### The TLCd is neurochemically distinct

**Fig. 6:**
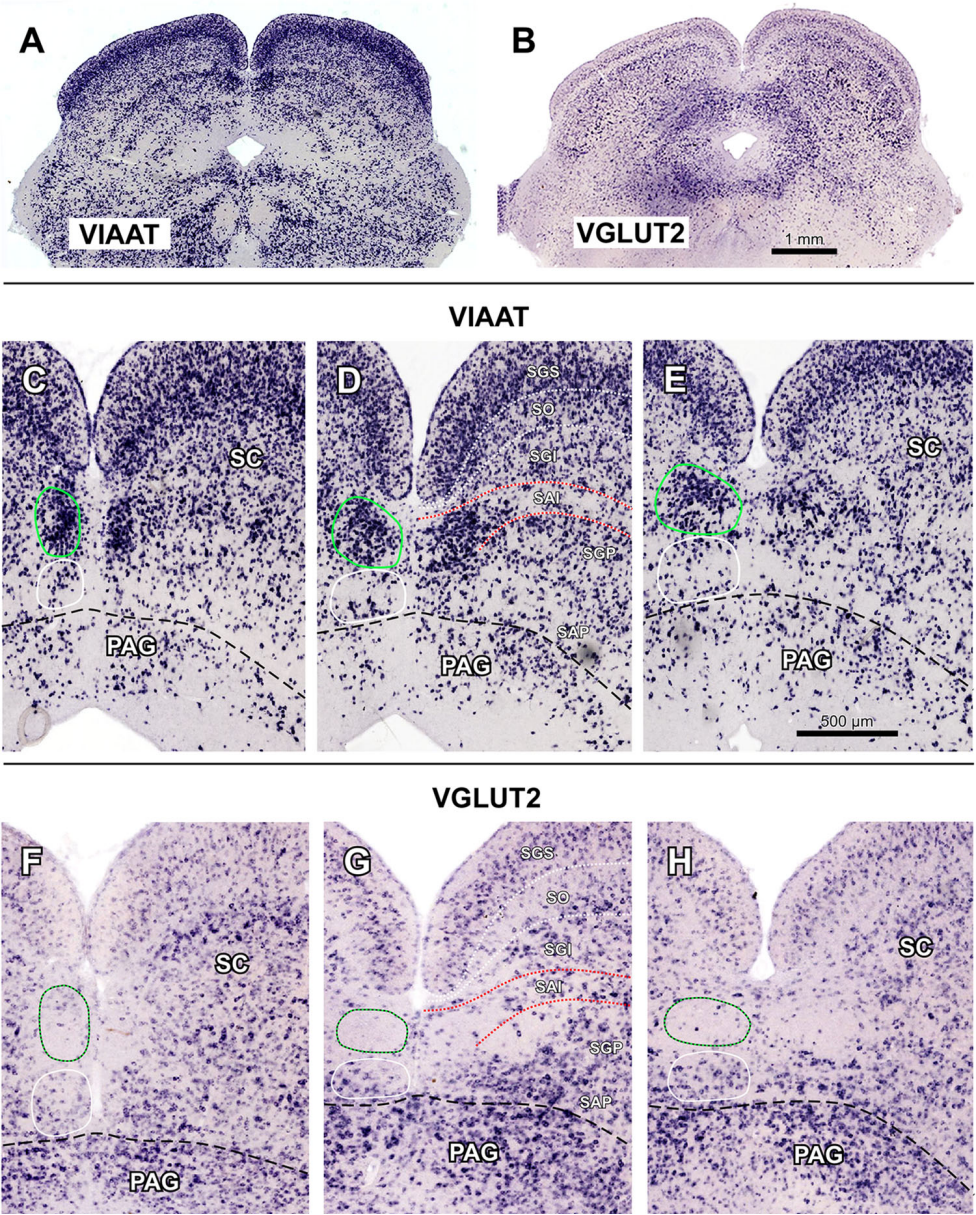
Rat TLCd neurons express the mRNA for VIAAT, but not for VGLUT2. Micrographs of coronal sections of the rat midbrain tectum processed by in situ hybridization to visualize the expression of VIAAT (**a**, **c**–**e**) or VGLUT2 (**b**, **f**–**h**). **a** and **b** show panoramic views of sections through the central rostrocaudal third of the TLCd. **c**–**h** show details of sections through the caudal (**c**, **f**), central (**d**, **g**) and rostral (**e**, **h**) portions of the TLCd. On the *left side*, the TLCd has been outlined in *green*. Note the complementary pattern of staining in the TLCd with the two markers. The *horizontal band* in the *center* of the SC with a higher concentration of neurons labeled for the VIAAT, visible in **a**, **c**–**e**, corresponds to the *upper portion* of the *deep gray layer* (SGP); the SAI stands out due to the paucity of labeled neurons, which strongly contrasts with the abundance of somata in the TLCd. *Calibration bar* in **b** applies also to **a**. *Calibration bar* in **e** applies also to remaining panels. *Calibration bars* uncorrected for shrinkage

The TLCd of the rat is readily appreciated in sections processed by in situ hybridization to reveal the expression of the mRNA for the vesicular inhibitory amino acid transporter (VIAAT). In all sections through the TLCd, the nucleus stands out due to the extremely high concentration of labeled cell bodies (Fig. 6a, c–e), which contrasts with the paucity of labeled neurons in the ventrally located TLCv. Indeed, the vast majority of, if not all, TLCd neurons express the VIAAT. The density of labeled neurons is conspicuously higher in the TLCd than in the laterally located intermediate and deep layers of the SC. This difference is particularly striking when one compares the TLCd with the SAI, which contains a very low concentration of labeled neurons (Fig. 6a, c–e).

Contrary to the expression of VIAAT, in sections processed to visualize the mRNA for the vesicular glutamate transporter 2 (VGLUT2), the TLCd stands out due to the virtual absence of labeled neurons, which contrasts with the abundance of labeled neurons in the TLCv and in the intermediate and deep layers of the SC (Fig. 6b, f–h). Taken together, the expression patterns of VIAAT and VGLUT2 stress the identity of the TLCd as a separate nucleus composed almost exclusively of presumably GABAergic neurons.

In sections processed to visualize the mRNA for the vesicular glutamate transporter 1 (VGLUT1), all tectal nuclei (including the TLCd, TLCv, and SC) are completely devoid of labeling (not shown).

### The TLCd is hodologically distinct

To confirm the identity of the TLCd and gain an initial insight into its function, we analyzed the neural connections of the nucleus in two steps. We first injected the sensitive bidirectional tracer BDA into the rat TLCd to unravel the sources of input to the nucleus, and to pinpoint the main targets of its efferent projections. As described below, the main target of the TLCd appears to be the ipsilateral lateral complex of the thalamus, which includes the lateral posterior (LP) and laterodorsal (LD) nuclei. We then injected the tracer FluoroGold (FG) into the lateral thalamic complex to retrogradely label TLCd neurons.

#### Cases with injection of BDA into the TLCd

**Fig. 7:**
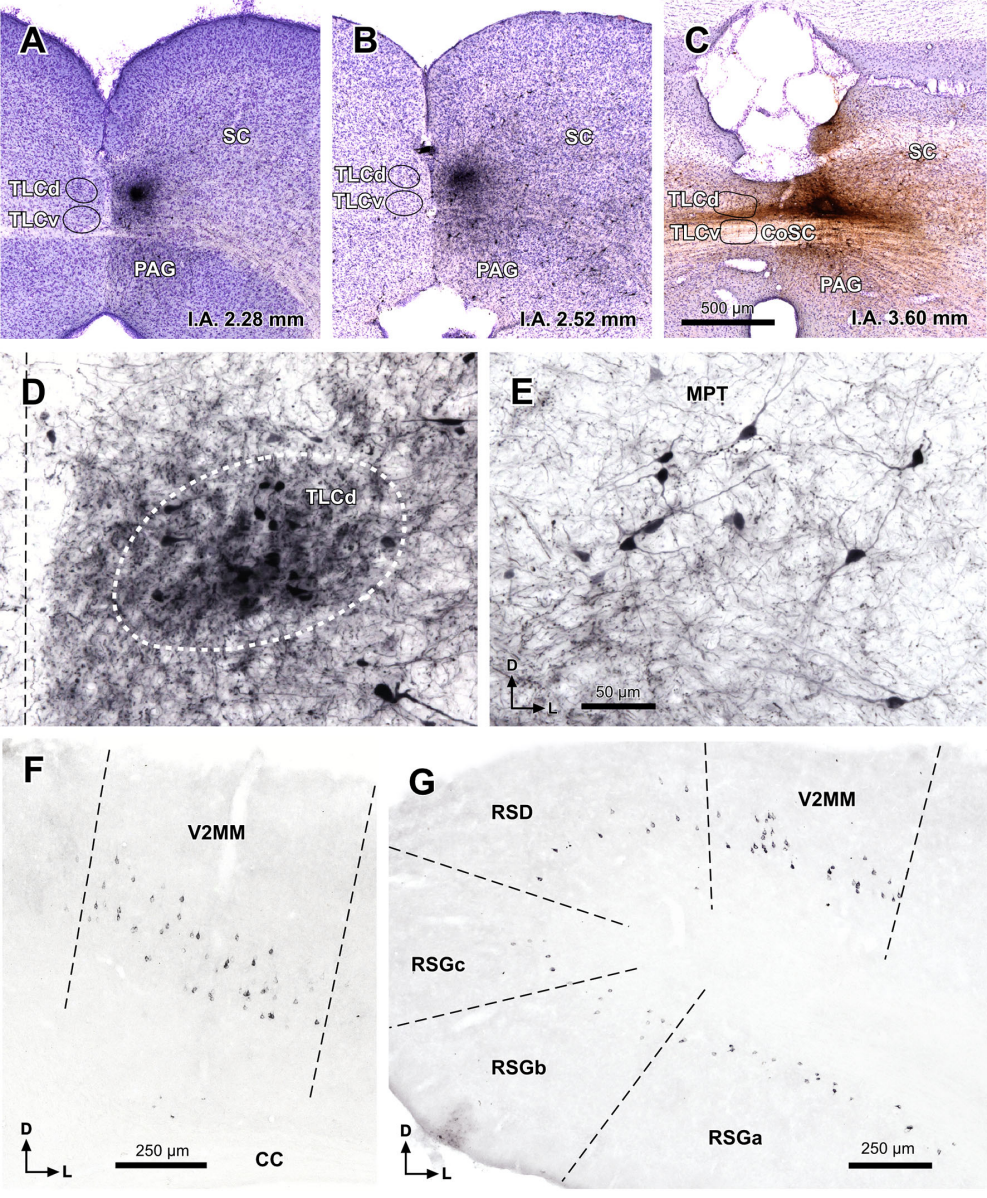
Main sources of input to the rat TLCd. **a–c** Micrographs of coronal sections through the center of single injection sites of BDA in the central (**a**, case 01101; **b** case 02088) or the rostral (**c** case 00268) rostrocaudal third of the TLCd. **d** Numerous TLCd neurons are retrogradely labeled in a section located 1,280 μm more caudal than the injection site depicted in **c**. **e** Retrogradely labeled neurons in the ipsilateral MPT (case 00268). **f** Retrogradely labeled neurons in a section through the rostral region of the ipsilateral V2MM (case 02088). **g** Retrogradely labeled neurons in a caudal section through the ipsilateral V2MM and retrosplenial cortices (case 02088). *Calibration bar* in **c** applies also to **a** and **b**. *Calibration bar* in **e** applies also to **d**. For abbreviations, see list

The following description is based on five cases with small, single injection sites (maximum diameter ≤250 μm) confined to the TLCd and placed at different rostrocaudal levels of the nucleus (Fig. 7a–c). The results of all cases were qualitatively similar. However, the number of labeled neurons and fibers was higher in the cases with more rostrally located injection sites.

In each case, retrogradely labeled neurons were found within the TLCd at levels more caudal than the injections site (Fig. 7d), but not at more rostral levels, which suggests that the axons of TLCd projection neurons span the nucleus longitudinally and exit it rostrally as part of the fascicle of the TLCd. It also suggests that TLCd neurons may be contacted by other TLCd neurons located more caudally, but not the other way around.

In every experiment, BDA-labeled cell bodies were found outside the TLCd in a large variety of neural centers, from the caudal medulla to the telencephalon. Likewise, labeled axons were found in various locations, from the caudal medulla to the rostral thalamus. The detailed analysis of the neural connections of the TLCd exceeds the aims of this article, which is concerned with the identity of the nucleus. For comparison with the projections of the adjacent TLCv and SC, it is noteworthy that TLCd projections are largely ascending and predominantly ipsilateral, and that TLCd axons do not contribute to the predorsal bundle or any of the descending pathways that constitute the main outflow of the intermediate and deep layers of the SC (see “Discussion”). Therefore, we focus below on what appears to be the main sources of input to the TLCd and the main targets of TLCd projections.

Numerous labeled neurons were found in the medial pretectal nucleus (MPT) and in vision-related areas of the cerebral cortex [secondary visual mediomedial area (V2MM) and granular and dysgranular retrosplenial cortices] (Fig. 7e–g), suggesting that these neural centers are major sources of input to the TLCd. Most retrogradely labeled neurons displayed a punctate reaction product that filled only the cell body and the proximal portion of the dendrites.

In the MPT ipsilateral to the injection site, labeled neurons were widely distributed throughout the nucleus and possessed small oval or polygonal somata (maximum diameter 10–15 μm) (Fig. 7e). Many neurons had their main axis oriented horizontally. The contralateral MPT contained very few labeled neurons.

In the cerebral cortex, regardless of the area, labeled neurons displayed a typical pyramidal morphology and were systematically located in layer V (Fig. 7f–g). In the V2MM, labeled neurons were found bilaterally, with a clear ipsilateral predominance. On the contralateral side, neurons were found mostly in the rostrocaudal central third of the V2MM. However, ipsilaterally, labeled layer V pyramidal neurons extended throughout the entire rostrocaudal extent of V2MM, and even beyond the rostral limit of V2MM into the cortical area identified in the atlas of Paxinos and Watson (2005) as the medial parietal association cortex.

In the retrosplenial cortices, labeled neurons were found only in sections more caudal than the rostral pole of the SC. Ipsilaterally, neurons were particularly abundant through all three regions of the retrosplenial granular cortex (regions a, b and c), and much scarcer in the retrosplenial dysgranular cortex (Fig. 7g). Contralaterally, labeled neurons were much scarcer and were scattered throughout the same areas.

**Fig. 8:**
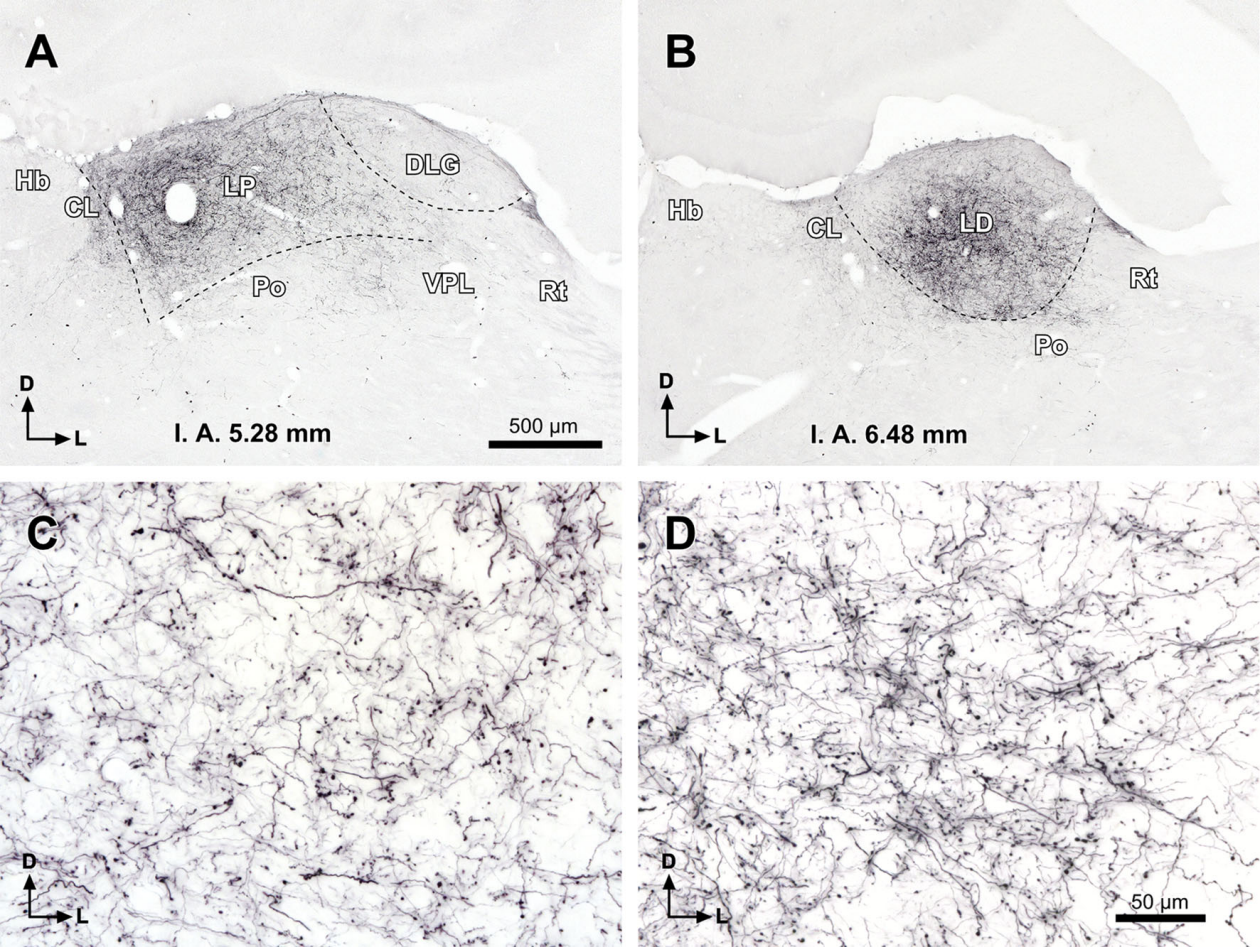
The TLCd sends dense projections to the ipsilateral lateral complex of the thalamus. Micrographs of coronal sections showing dense terminal fields of fibers labeled in the LP (**a**) and LD (**b**) following a single injection of BDA into the ipsilateral TLCd (case 00268; see injection site in Fig. 7c). **c**, **d** Details of the terminal fields of TLCd fibers from the sections depicted in **a** and **b**, respectively. *Calibration bar* in **a** applies also to **b**. *Calibration bar* in **d** applies also to **c**. *Calibration bars* uncorrected for shrinkage. For abbreviations, see list

In all cases with injections of BDA confined to the TLCd, labeled fibers left the nucleus rostrally via the fascicle of the TLCd, to cross the pretectum. No axonal bundles were labeled in the adjacent SAI (Fig. 7a, b). The densest terminal field was found throughout the ipsilateral lateral thalamic complex, spanning without interruption the caudally located LP and the rostrally located LD (Fig. 8). This terminal field was remarkable due to its density, unexpectedly high for such small injection sites, and due to the abundance and relatively small size of en passant and terminal varicosities, presumed to represent synaptic specializations (Fig. 8c, d). The contralateral lateral thalamic complex received a comparatively weak projection, which seemed to concentrate at rostral levels.

#### Cases with injection of FG into the lateral complex of the thalamus

**Fig. 9:**
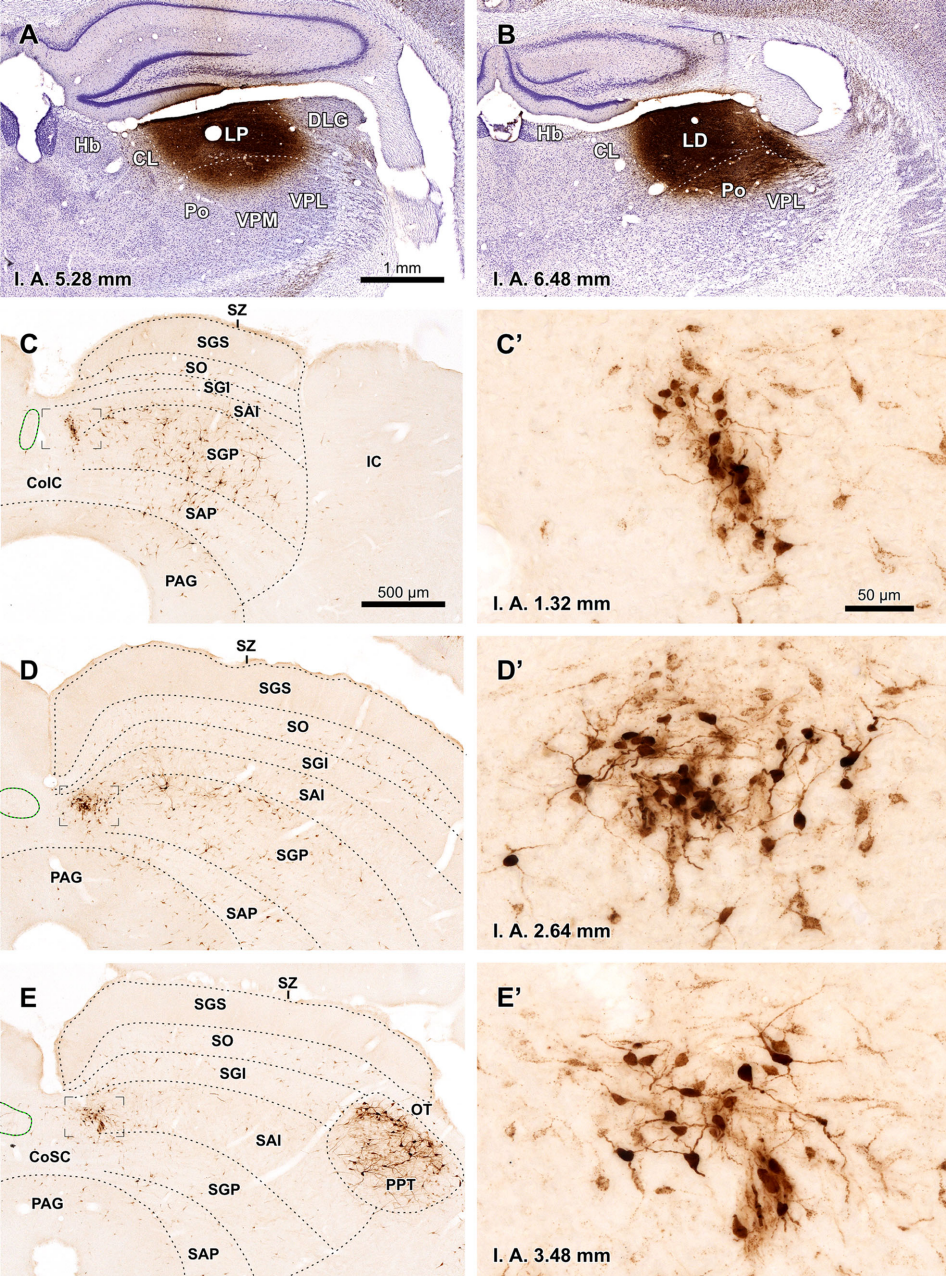
TLCd neurons are retrogradely labeled following injections of FluoroGold into the ipsilateral lateral complex of the thalamus. **a**, **b** Micrographs of Nissl-counterstained coronal sections through the center of the injection sites of FluoroGold into LP (**a**) and LD (**b**). Case 05239. **c**–**e** Micrographs of coronal sections through various rostrocaudal levels of the midbrain tectum of the same case. At each level, the TLCd stands out as a darkly stained spot whose density of labeled neurons surpasses that of any region of the SC. **c′–e′** Details of the TLCd in the same sections shown in the *left column*. The interaural (IA) coordinate of each plane is indicated at the *bottom* of the micrographs. *Calibration bar* in **a** applies also to **b**. *Calibration bar* in **c** applies also to **d**, **e**. *Calibration bar* in **c′** applies also to **d′**, **e′**. *Calibration bars* uncorrected for shrinkage. For abbreviations, see list

The present description is based on 12 cases with unilateral deposits of FG into the lateral complex of the thalamus. In an attempt to maximize the labeling of TLCd neurons, in each of three cases we made two caudal injections centered in LP and two rostral injections centered in LD. At each level, one injection was placed 300 μm more lateral than the other. The resulting combined injection site covered most of the lateral complex of the thalamus, encroaching very little upon neighboring structures (e.g., Fig. 9a, b).

**Fig. 10:**
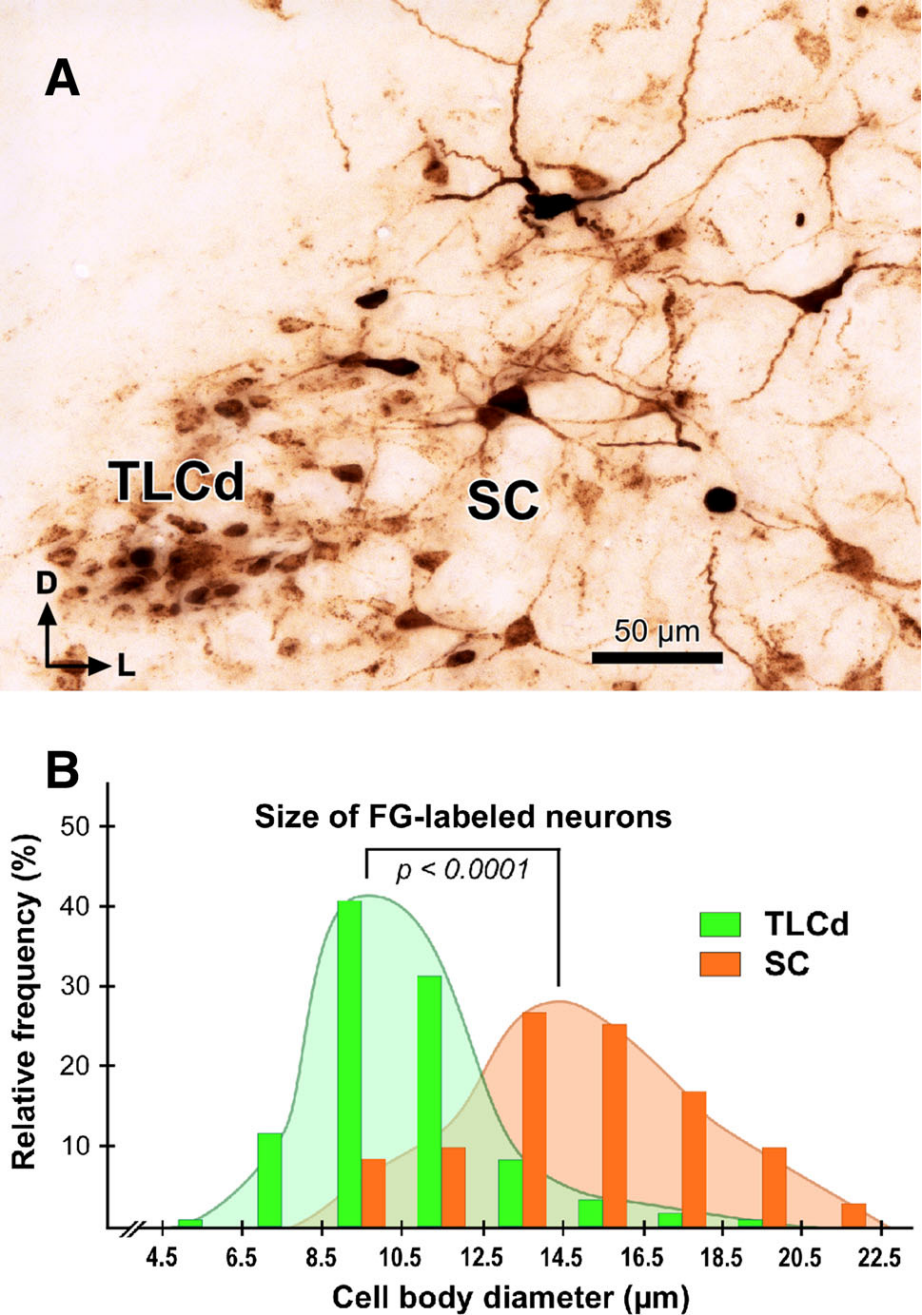
Comparison between TLCd and SC neurons that innervate the lateral complex of the thalamus. **a** High magnification micrograph of a section through the central rostrocaudal third of the TLCd from a case similar to the one depicted in Fig. 9. Case 05240. Labeled TLCd neurons (left half of the micrograph) are small, tightly packed, posses homogeneous round or oval cell bodies, and display a punctate reaction product limited to the cell body and, in some instances, the most proximal portions of their dendritic trees. In contrast, labeled SC neurons possess much larger, polygonal cell bodies and display an impressive filling of their dendritic trees. *Calibration bar* uncorrected for shrinkage. **b** Histogram of the maximum diameter of the cell body of neurons labeled in the TLCd (*green* mean = 10.64 μm ± 2.24 SD; *n* = 120) and the adjacent SC (*orange* mean = 14.90 μm ± 2.98 SD; *n* = 71) measured from the case depicted in Fig. 9. Measured SC neurons were limited to those located within 500 μm of the lateral border of the TLCd. Note that the labeled SC neurons are on average much larger and more heterogeneous than the labeled TLCd neurons

In addition to the expected labeling in all nuclei known to innervate the lateral complex of the thalamus (reviewed by Groenewegen and Witter 2004), abundant neurons were labeled in the TLCd ipsilateral to the injection site (Fig. 9c–e). In every section, labeled neurons were widely distributed throughout the nucleus and displayed a wide range of labeling intensities, from neurons with faint punctate labeling confined to the cell body, to neurons with dense, diffuse labeling that filled the dendrites (Figs. 9c′–e′, 10a). Altogether, it seemed that most TLCd neurons were labeled. This dense retrograde labeling allowed the unequivocal distinction of the TLCd from the adjacent SC and TLCv (Fig. 8c–e). Figure 10a illustrates the differences in the size and shape of the cell bodies and in the pattern of dendritic filling between neurons labeled in the TLCd and those labeled in the adjacent SAI. Morphometric analysis of the labeled neurons demonstrated that TLCd neurons were significantly smaller (Fig. 10b). Only rarely did the TLCd contralateral to the injection site contain labeled neurons.

**Fig. 11:**
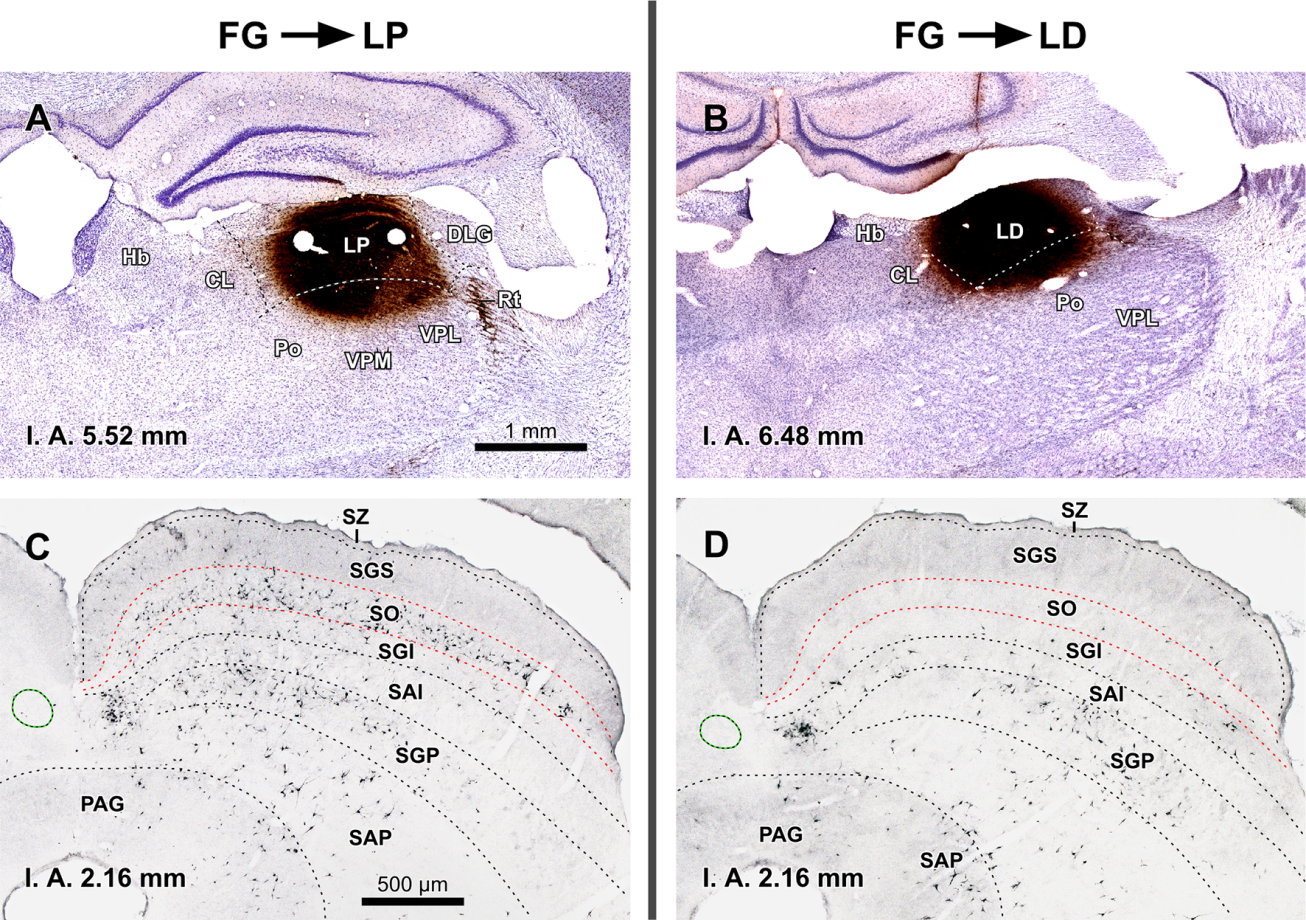
TLCd neurons innervate both LP and LD. **a**, **b** Micrographs of Nissl-counterstained coronal sections through the center of a single injection site of FluoroGold into LP (**a** case 05222) or LD (**b** case 05209). **c**–**d** Micrographs of coronal sections through the ipsilateral half of the midbrain tectum of the same cases. **c** Following injection into LP, numerous neurons are labeled in the optic layer (SO), with fewer neurons labeled in deep layers, and even fewer in the intermediate layers. The neurons labeled in the superficial gray layer (SGS) are the consequence of the spread of the tracer into the most medial portion of the dorsal lateral geniculate nucleus (DLG). **d** Following injection into LD, labeled neurons are scattered throughout the deep layers of the SC and, to a lesser extent, in the intermediate layers, whereas the superficial layers are devoid of labeled neurons. In both types of cases, the concentration of neurons labeled in the TLCd surpasses that of any other territory of the tectum. The interaural (IA) coordinate of each plane is indicated at the *bottom* of the micrographs. *Calibration bar* in **a** applies also to **b**. *Calibration bar* in **c** applies also to **d**. *Calibration bars* uncorrected for shrinkage. For abbreviations, see list

Projections from the SC to LP and the projections from the SC to LD have been reported to originate in different populations of SC neurons (see references below). Therefore, we compared cases with single injections of FG into LP (Fig. 11a) with cases with single injections into LD (Fig. 11b) to investigate whether there was a difference in the morphology or distribution of the neurons labeled in the TLCd. In cases with injections into LP, there were abundant labeled neurons in the optic layer (stratum opticum) of the ipsilateral SC, with fewer neurons labeled in more ventral layers (Fig. 11c), thus confirming previous reports (Donnelly et al. 1983; Sugita et al. 1983; Lane et al. 1993, 1997; Hilbig et al. 2000; Masterson et al. 2009). In cases with injections into LD, labeled neurons were scattered throughout the intermediate and deep layers of the ipsilateral SC, and the optic layer was virtually devoid of labeled neurons (Fig. 11d), which is consistent with previous results (Thompson and Robertson 1987). In both types of cases, abundant neurons were labeled throughout the rostrocaudal extent of the ipsilateral TLCd and they showed similar morphology and distribution (Fig. 11c–d).

### Summary of results

Our data demonstrate that the TLCd is a distinct nucleus of the mammalian brain. It differs from the surrounding nuclei (TLCv and SC) by its cytoarchitecture, myeloarchitecture, neurochemistry and neural connections. The TLCd of the rat consists of a homogeneous population of small, presumably GABAergic neurons that receive input from the pretectum and from secondary visual and retrosplenial cortices, and provides the LP and LD with a dense inhibitory projection.

## Discussion

### The TLCd is a distinct nucleus

The present study represents a systematic investigation of a previously uncharacterized nucleus of the mammalian brain: the TLCd. Although the existence of the TLCd was noted in the course of a methodical investigation of the ventrally adjacent TLCv (Saldaña et al. 2007), herein we employed a constellation of anatomical techniques to demonstrate that the TLCd is indeed a distinct nucleus of the rat brain. Despite its narrow appearance in coronal sections, the size of this longitudinally oriented structure is impressive, as the nucleus spans the entire rostrocaudal length of the SC.

It may seem surprising that a nucleus as large as the TLCd has remained unnoticed until now. The border between TLCd and the SC, although distinct at close inspection (e.g., Figs. 3, 4), may seem ill-defined at first glance, which explains why, in the relatively scarce published works whose illustrations contain the midline, the territory occupied by the TLCd has been traditionally included in the most medial part of the SC (e.g., Olszewski and Baxter 1954; Berman 1968; Swanson 1999; Hof et al. 2000; Paxinos and Watson 2005). Unfortunately, considerable confusion burdens the literature pertaining to the stratification of the paramedian region of the tectum, and the territory occupied by the TLCd has been non-systematically assigned to the medial portion of the intermediate gray layer (e.g., Rieck et al. 1986 [cat]; Swanson 1999 [rat]; Meredith et al. 2001 [ferret]; Helms et al. 2004 [rat]; Bajo et al. 2010 [ferret]), the SAI (Redgrave et al. 1986, 1990 [rat]; Rhoades et al. 1986 [hamster]; Harvey and Worthington 1990 [rat]; Harvey et al. 2001 [rat]; Favaro et al. 2011 [rat]), both intermediate layers (García del Caño et al. 2000, 2001 [rat]) or the deep layers (Ma et al. 1990 [monkey]; Furigo et al. 2010 [rat]), or even left outside of any identifiable layer (e.g., Huerta and Harting 1984 [cat]; Matsuyama and Kawamura 1985 [rat]; Paxinos and Watson 2005 [rat]; Comoli et al. 2012 [rat]). As discussed below, our data unequivocally demonstrate that the TLCd is located immediately medial to the SAI.

Another reason that the TLCd may have gone unnoticed is that many commonly used neurochemical markers do not highlight the nucleus. For example, in sections of the rat midbrain stained for parvalbumin, calbindin, calretinin, calcitonin gene-related peptide, neurofilament protein (SMI–32), tyrosine hydroxylase, NADPH-diaphorase, acetylcholinesterase or adenosine deaminase, the TLCd is hardly distinguishable (Miguel-Hidalgo et al. 1989; Lane et al. 1993; Paxinos et al. 1999).

**Fig. 12:**
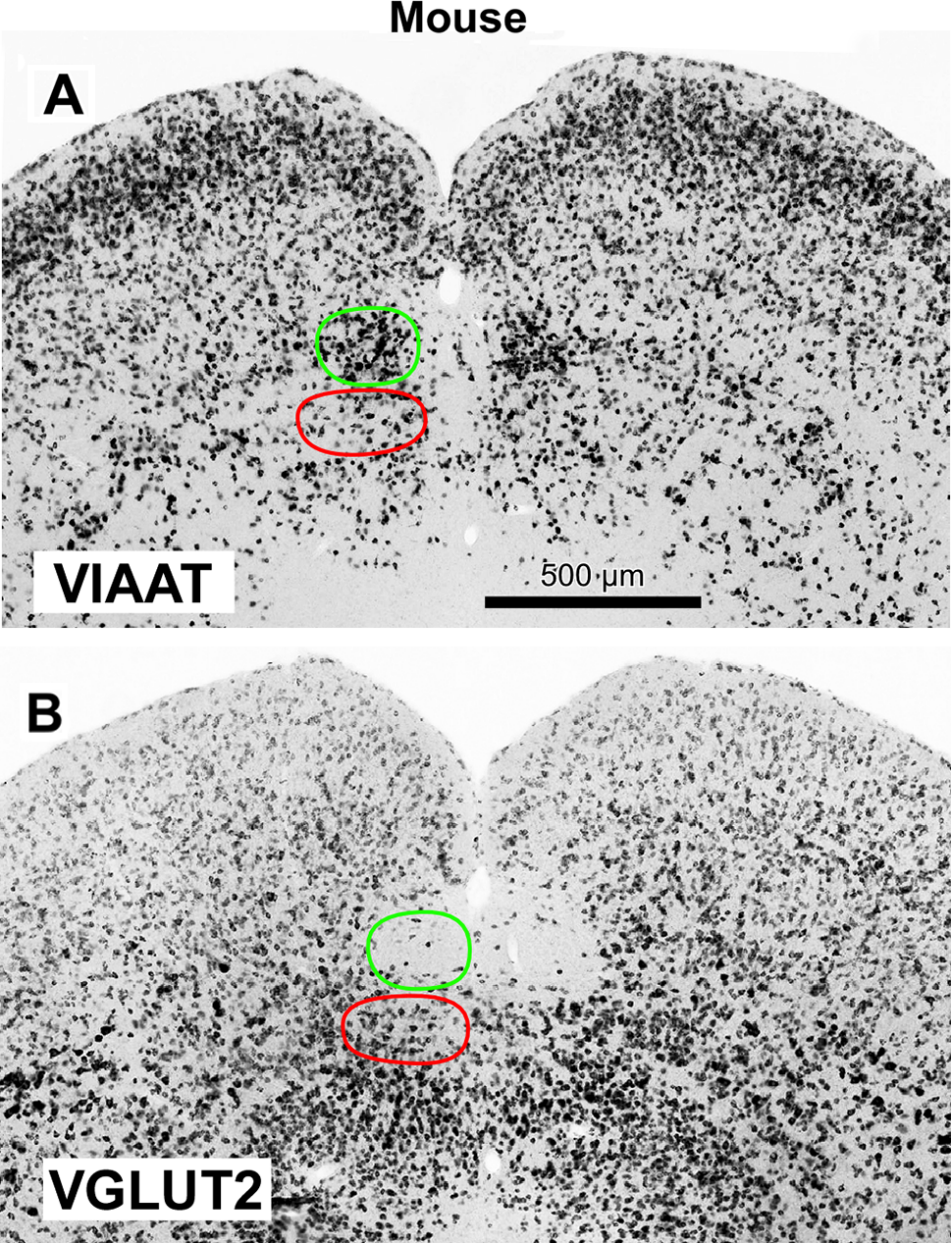
Mouse TLCd neurons express the mRNA for VIAAT, but not for VGLUT2. Micrographs of coronal sections of the mouse midbrain tectum processed by in situ hybridization to visualize the expression of the mRNA for VIAAT (**a**) or the mRNA for VGLUT2 (**b**). Like in the rat (Fig. 6), the mouse TLCd stands out due to the very high density of neurons that express the VIAAT and the paucity of neurons that express the VGLUT2. *Calibration bar* in **a** applies also to **b**. *Calibration bar* uncorrected for shrinkage

Some reports in the literature did identify important features of the TLCd, adding strength to its present characterization. A continuous, longitudinal pool of neurons strongly immunoreactive for glutamic acid decarboxylase (GAD), tentatively called “area commissuralis colliculi superioris et inferioris”, was reported by Mugnaini and Oertel (1985) in the paramedian region of the rat midbrain tectum (see their Fig. 102), in a location consistent with TLCd. Similarly, a distinct cluster of neurons expressing the mRNA for GAD was reported in the same location by Harvey et al. (2001; their Figs. 2D, 3D). Furthermore, an unmistakable cluster of brightly fluorescent neurons was found in the location of the TLCd in a line of transgenic mice expressing the fluorescent protein Venus under the control of the VIAAT promoter (Wang et al. 2009; their Fig. 6). The latter observation is particularly relevant given that our own material indicates that the pattern of expression of the VIAAT in the tectum of the mouse is virtually identical to that of the rat (compare Figs. 6 and 12). All these data strongly suggest that in both the mouse and the rat most, if not all, TLCd neurons are GABAergic. Finally, the TLCd stands out due to the presence of neurons and fibers immunoreactive for leu-enkephalin (Miguel-Hidalgo et al. 1990; see their Fig. 2B, E), and to the high levels of expression of the alpha-7 nicotinic acetylcholine receptor in developing and mature rats and mice (Happe and Morley 2004; see their Figs. 1, 2).

The existence of the TLCd is also supported by several previous tract-tracing studies. Following injection of BDA into the retrosplenial cortex of the rat, a distinct cluster of terminal fibers was labeled in the territory occupied by the TLCd in both normal rats (García del Caño et al. 2001; Tsumori et al. 2001) and in rats that previously underwent neonatal enucleation (García del Caño et al. 2001). Likewise, the injection of peroxidase-conjugated wheat-germ agglutinin (WGA-HRP) into secondary visual areas of the rat neocortex resulted in a high concentration of labeled fibers in the territory occupied by the ipsilateral TLCd (Harvey and Worthington 1990; see their Fig. 5A). Moreover, numerous neurons were labeled in the location of the TLCd ipsilateral to injections of HRP into the laterodorsal thalamic nucleus (LD) of the rat (Thompson and Robertson 1987; their Figs. 3, 5), deposits of gold-WGA-HRP which involved the lateral posterior thalamic nucleus (LP) of the rat (Carstens et al. 1990; their Fig. 6D), and injections of fluorescein-conjugated dextran amine in the pulvinar nucleus of the cat (Baldauf et al. 2005; their Fig. 1F), the feline homolog of the rodent LP. All these findings lend firm support to the connections of the TLCd described in the current study and to the hypothesis that it is present not only in rodents, but also in other mammals. Indeed our preliminary observations suggest that the cytoarchitectural criteria that define the TLCd of the rat allow the identification of a comparable nucleus in the paramedian region of the tectum of other mammalian species, including lagomorphs, carnivores, non-human primates and humans.

#### Differences between the TLCd and the TLCv

Despite their close apposition and their organizational similarities, the TLCd and the TLCv differ in various fundamental aspects. *Anatomically* The TLCv, but not the TLCd, is traversed by the commissure of the IC and the commissure of the SC. Conversely, the TLCd, but not the TLCv, is spanned by a conspicuous fascicle of longitudinally oriented axons. *Cytoarchitecturally* The TLCd lacks for the most part neurons comparable to medium-sized neurons of the TLCv, and TLCd neuronal somata are significantly smaller and more tightly packed than those of TLCv small neurons (Saldaña et al. 2007). *Neurochemically* The expression of amino acid vesicular transporters (Figs. 6, 12) suggests that most, if not all, TLCd neurons are GABAergic, whereas the TLCv may contain a mixture of GABAergic and glutamatergic neurons. *Hodologically* The projections of the TLCd are predominantly ascending, while those of the TLCv are mostly descending. More specifically, the TLCd, but not the TLCv, innervates the lateral complex of the thalamus (Figs. 8, 9, 10, 11); conversely, the TLCv, but not the TLCd, is innervated by the IC (Aparicio et al. 2010) and is reciprocally connected with the superior paraolivary nucleus (Viñuela et al. 2011). Therefore, while the TLCv is connected with auditory centers, the connections of the TLCd appear to be related to the visual system. And finally, *electrophysiologically* TLCv neurons respond to acoustic stimulation, whereas TLCd neurons do not respond to sound (Saldaña et al. 2007; Marshall et al. 2008).

#### Differences between the TLCd and the SC

A pivotal question in establishing the identity of the TLCd is to determine whether it represents a separate nucleus or rather a more or less specialized region of the SC, and particularly a region of the SAI, in which it has been often included. This is more than just a semantic issue, as recognizing the TLCd as a distinct nucleus will influence future strategies designed to investigate it because different nuclei may have different ontogenetic origins and subserve different functions.

Our results demonstrate that the TLCd differs conspicuously from the adjacent regions of the SC in terms of *cytoarchitecture* TLCd neurons are remarkably homogeneous, rostrocaudally oriented, and significantly smaller and more tightly packed than neighboring SC neurons. In fact, these differences give rise to the sharp border between the TLCd and the SC that is readily appreciated in many sections (e.g., Figs. 3, 4). The TLCd also has different *myeloarchitecture* The longitudinal fibers that span the TLCd and which form the fascicle of the TLCd differ from the longitudinal fibers of the SAI in their thickness and their pattern of aggregation into bundles (Fig. 5).

One of the strongest arguments in favor of the TLCd as a separate entity is the proportion of GABAergic neurons it contains. According to our results, in both rats and mice TLCd neurons are presumably GABAergic because they express the VIAAT, whereas in the SAI the proportion of neurons that express the VIAAT is low. In the rat, fewer than 5 % of neurons in the SAI are GAD-immunopositive, which makes it the SC layer with the lowest concentration of GABAergic neurons (Mugnaini and Oertel 1985; see their schematic drawings A 2.2 through P 0.1). In contrast, 50–90 % of neurons in the TLCd are GAD-immunopositive, a proportion unmatched by any SC region (Mugnaini and Oertel 1985; their schematic drawings A 2.2 through P 0.5).

The TLCd and the SC differ also in their neural connections. As shown by our experiments, the main target of the TLCd is the ipsilateral lateral complex of the thalamus. Two aspects of this projection are particularly noteworthy: first is the very high density of the projection, which one would not expect from a nucleus as narrow as the TLCd, especially considering the small size of our tracer injection sites; and second, the fact that the TLCd innervates with similar density both the LP and the LD. This is clearly different from the tectothalamic projections of the SC, which are known to innervate densely LP, but not LD. In our own experiments, many fewer neurons were labeled in the SC following injections of retrograde tracers in LD than in cases with similar injections centered in LP (Fig. 11). Moreover, our unpublished cases with injections of BDA in regions of the SC lateral to the TLCd reveal a dense projection to LP, whereas the projection to LD appears very weak, much weaker than the one revealed by the much smaller injections into TLCd.

A further difference between the tectothalamic projections of the TLCd and those of the SC may be related to the neurotransmitters released at their synapses. The synaptic boutons labeled in LP following injections of the anterograde tracer PHA-L into the rat SC exhibit ultrastructural features usually associated with excitatory neurotransmission and are immunoreactive for the vesicular glutamate transporter 2 (VGLUT2), which has prompted the suggestion that VGLUT2 may be used as a specific marker for the SC axons that innervate LP (Masterson et al. 2009). This is particularly meaningful given that, according to our data, in both the rat and the mouse TLCd neurons do not express VGLUT2. Given the likely GABAergic nature of TLCd neurons, one would expect its synaptic boutons in LP (and LD) to be very different from colliculothalamic boutons.

**Fig. 13:**
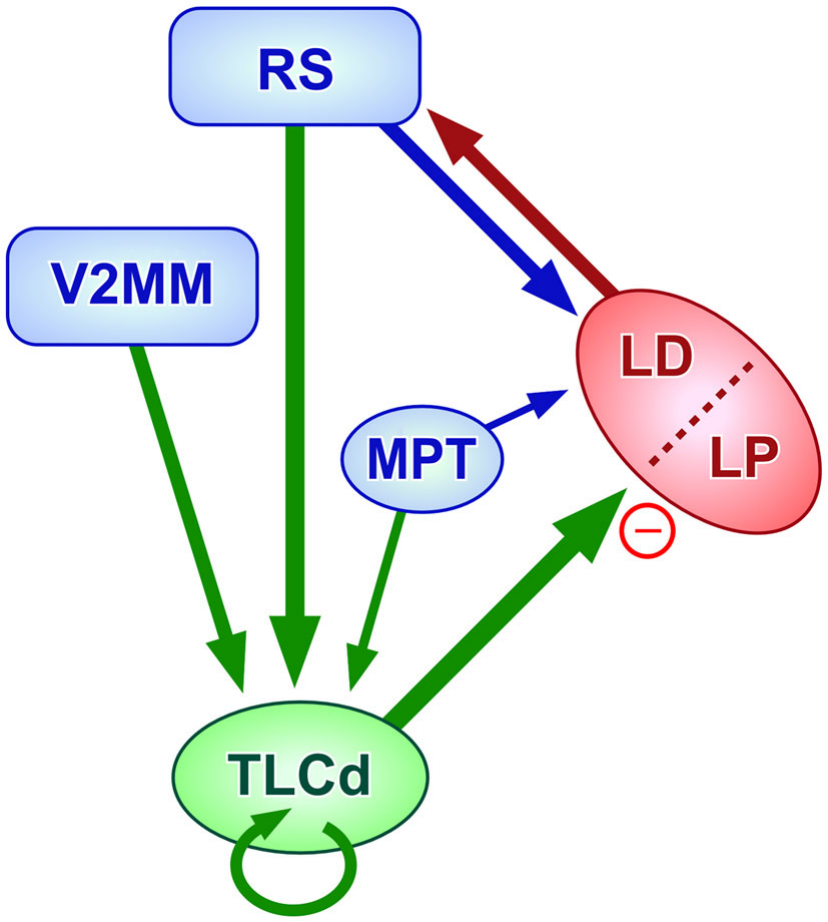
Schematic diagram of the main neural connections of the rat TLCd. The afferent and efferent connections of the TLCd unraveled in this study have been indicated by *green arrows*. The thickness of the different arrows roughly corresponds to the density of the projections. Note that the TLCd sends a dense inhibitory projection to LP and LD

Another major hodological difference between the TLCd and the SC is that the TLCd lacks the descending, predominantly crossed projections to brainstem and spinal centers involved in the control of eye, head, pinna or body movements, which are characteristic of the intermediate and deep layers of the SC (reviewed by May 2006). In our cases with injections of BDA into the TLCd, the paramedian pontine reticular formation, which is the main target of the descending predorsal bundle, was virtually devoid of labeled fibers. This observation is consistent with previous tract-tracing investigations: following injections of retrograde tracers into the tecto-recipient zones of the paramedian reticular formation, labeled SC neurons were concentrated in the contralateral SAI, but always at a distance from the midline, so no labeled neurons were found within the TLCd (e.g., Redgrave et al. 1986 [rat], see their Fig. 1; Redgrave et al. 1990 [rat], their Fig. 1; May 2006 [cat, macaque], his Fig. 13).

All these arguments strongly suggest that, just like the TLCv, the TLCd is not part of the SC. The organization of these two paramedian columns is clearly different from the alternating horizontal layers of gray and white matter of the SC. Therefore, even if the TLCd and the TLCv are anatomically considered “within” the SC, one should keep in mind that they are not actually part of the horizontal layered system of the SC, nor do they fit the mosaic-like pattern of vertical patches and modules found throughout the SC (Chevalier and Mana 2000; Harting 2004).

#### Three longitudinal columns in the paramedian region of mammalian midbrain tectum

The discovery of the TLCd and the TLCv reveals an unexpected level of longitudinal organization in the paramedian region of mammalian midbrain tectum. This region appears to be composed of three parallel, longitudinal columns: the TLCd, the TLCv, and the dorsomedial column of the periaqueductal gray matter (PAGdm), ordered from dorsal to ventral. While these three columns differ markedly in their cytoarchitecture, relationship with the tectal commissures, connections and electrophysiology, they may be related in other ways. For example, many PAGdm neurons possess dendrites that extend dorsally to enter the TLCv (Mantyh 1982; Beitz and Shepard 1985; Herrera et al. 1988). Similarly, the dendrites of many TLCv neurons extend dorsally or ventrally beyond the limits of the nucleus into the TLCd or the PAGdm (Herrera et al. 1988; Saldaña et al. 2007). Finally, the TLCd contains neurons whose dendrites make their way down into the TLCv (our unpublished observations). This intriguing arrangement raises interesting questions as to whether the three columns are ontogenetically related, share incoming information and/or participate in coordinated functions.

### What is the role of the TLCd?

Despite the limited information currently available about the TLCd, our results provide a conceptual framework for future studies. The findings presented herein, coupled with our unpublished observations that the TLCd is present in species as different as rodents, lagomorphs, carnivores and primates, suggests that it may be common to all terrestrial mammals, or even to the entire mammalian class. The function of the TLCd is, therefore, probably shared by a large variety of animals, and may extend to include non-mammalian vertebrates. For instance, due to its tectal location and longitudinal orientation, the prominent torus longitudinalis of the fish brain (e.g., Folgueira et al. 2007) resembles the paramedian columns of the mammalian tectum.

#### The TLCd as an extrathalamic source of inhibition to the lateral thalamus

Insight into the function of the TLCd can be gained from the fact that its neurons are most likely GABAergic and send their axons to LP and LD, two higher order thalamic nuclei commonly included as part of the visual system. In the LP of the rat, GABAergic terminals represent 10–20 % of all synaptic boutons (Çavdar et al. 2011). The most conspicuous source of GABAergic innervation of thalamic neurons is the reticular thalamic nucleus (e.g. Wang et al. 2001 [cat]; Wanaverbecq et al. 2008 [rat]). Local thalamic GABAergic interneurons are unlikely to account for a significant percentage of the GABAergic terminals because GABAergic neurons are exceedingly rare in the thalamus of the rat (Arcelli et al. 1997). Other sources of GABAergic projections to higher order thalamic nuclei have been identified in recent years. In the rat, the zona incerta sends widespread GABAergic projections to several higher order thalamic nuclei, but interestingly such inputs seem to spare for the most part LP and LD (Barthó et al. 2002). These two nuclei are, however, innervated by GABAergic neurons of the anterior pretectal nucleus (Bokor et al. 2005; see Baldauf et al. 2005 for related data in the cat) whose axons form large terminals that establish multiple synapses with their postsynaptic neurons (Bokor et al. 2005; Wanaverbecq et al. 2008). Although the ultrastructure of the synapses between boutons of TLCd axons and LP and LD neurons has not been studied, our tract-tracing data suggest that TLCd boutons are smaller than those of the anterior pretectal nucleus, and may be more similar to the boutons formed by axons of the reticular thalamic nucleus, which are also small and establish one single synapse with their targets (Wanaverbecq et al. 2008). Comparing the GABAergic thalamopetal projection of the anterior pretectal nucleus with that of the TLCd, there are two additional differences: first, while not all the pretectal neurons that innervate the thalamus are GABAergic, the TLCd apparently consists of a pure population of GABAergic tectothalamic neurons; and secondly, the anterior pretectal nucleus seems to exert a widespread effect over multiple thalamic nuclei, whereas the projections of the TLCd innervate very preferentially the lateral complex of the thalamus. Therefore, the TLCd may be unique as it constitutes the only know nucleus composed of GABAergic neurons dedicated to providing massive inhibition to higher order thalamic nuclei of a specific sensory modality.

#### Biological significance of the TLCd

Further insight into the role of the TLCd can be gained from its main connections, unraveled in this study and summarized in Fig. 13. In the rat, the TLCd is innervated by the medial pretectal nucleus (MPT), the secondary visual mediomedial area (V2MM) and the retrosplenial cortices, and sends dense projections to the lateral complex of the thalamus (LP and LD). Many of these structures are heavily interconnected (e.g., Thompson and Robertson 1987; Shibata 2000; Van Groen and Wyss 2003; Kamishina et al. 2009). In the rat, the retrosplenial cortices and the LD contain head-direction neurons, essential for spatial navigation (reviewed by Taube 2007), and have been implicated in learning and memory tasks with a visual component (Mizumori et al. 1994; Van Groen et al. 2002; Vann et al. 2009; Yoder et al. 2011). Moreover, V2MM has recently been shown to be critical for object-recognition memory (López-Aranda et al. 2009) and its development is promoted by the exposure to moving images (Sun et al. 2009). Therefore, the connections of the TLCd suggest that the function of the nucleus is related to the processing of hitherto unknown aspects of visual stimuli. Interestingly, in their study of the electrophysiological properties of TLCv neurons, Marshall et al. (2008) reported that TLCd neurons were unresponsive to sound or light. It is possible, however, that the visual stimuli used by these authors (a static flashlight or a laser pointer in a darkened sound-attenuated recording chamber) were inadequate to trigger the activity of TLCd neurons. Systematic investigations of the responses of TLCd neurons to varied visual stimuli will be necessary before the biological meaning of the nucleus can be understood. It would also be interesting to investigate to what extent the GABAergic innervation from the TLCd is involved in the processing of somatosensory information by LD neurons (Bezdudnaya and Keller 2008).

The TLCd represents an unexpected and promising focus for future research on modulation of thalamic activity and mammalian visual function. Knowledge about the nucleus is still rudimentary, but with currently available and newly emerging technologies, neuroscientists should be able to accomplish in a few years what took many decades to achieve in other nuclei of the brain. The anatomical results of the present study should aid in the design of future investigations.

## Abbreviations

Aq: Cerebral aqueduct
BDA: Biotinylated dextran amine
BIC: Brachium of the inferior colliculus
CL: Centrolateral thalamic nucleus
CoIC: Commissure of the inferior colliculus
CoSC: Commissure of the superior colliculus
DEPC: Diethylpyrocarbonate
DIG: Digoxigenin
DLG: Dorsal lateral geniculate nucleus
DpMe: Deep mesencephalic nucleus
GAD: Glutamic acid decarboxylase
Hb: Habenular complex
IA: Interaural
IC: Inferior colliculus
LD: Laterodorsal nucleus of the thalamus
LP: Lateral posterior nucleus of the thalamus
MPT: Medial pretectal nucleus
OT: Nucleus of the optic tract
PAG: Periaqueductal gray matter
PAGdm: Dorsomedial column of the periaqueductal gray matter
PB: Sodium phosphate buffer
pc: Posterior commissure
Po: Posterior thalamic nuclear group
PPT: Posterior pretectal nucleus
ReIC: Recess of the inferior colliculus
RePi: Recess of the pineal gland
RSD: Retrosplenial dysgranular cortex
RSGa: Retrosplenial granular cortex, a region
RSGb: Retrosplenial granular cortex, b region
RSGc: Retrosplenial granular cortex, c region
Rt: Reticular thalamic nucleus
SAI: Intermediate white layer (stratum album intermediale) of the superior colliculus
SAP: Deep white layer (stratum album profundum) of the superior colliculus
SC: Superior colliculus
SGI: Intermediate gray layer (stratum griseum intermediale) of the superior colliculus
SGP: Deep gray layer (stratum griseum profundum) of the superior colliculus
SGS: Superficial gray layer (stratum griseum superficiale) of the superior colliculus
SO: Optic layer (stratum opticum) of the superior colliculus
SZ: Zonal layer (stratum zonale) of the superior colliculus
TLC: Tectal longitudinal column
TLCd: Dorsal tectal longitudinal column
TLCv: Ventral tectal longitudinal column
V2MM: Secondary visual cortex, mediomedial area
VIAAT: Vesicular inhibitory amino acid transporter
VGLUT1: Vesicular glutamate transporter 1
VGLUT2: Vesicular glutamate transporter 2
VPL: Ventral posterolateral thalamic nucleus
VPM: Ventral posteromedial thalamic nucleus
WGA-HRP: Peroxidase-conjugated wheat-germ agglutinin
